# Reprogramming immunosuppressive myeloid cells by activated T cells promotes the response to anti-PD-1 therapy in colorectal cancer

**DOI:** 10.1038/s41392-020-00377-3

**Published:** 2021-01-08

**Authors:** Jing Chen, Hong-Wei Sun, Yan-Yan Yang, Hai-Tian Chen, Xing-Juan Yu, Wen-Chao Wu, Yi-Tuo Xu, Li-Lian Jin, Xiao-Jun Wu, Jing Xu, Limin Zheng

**Affiliations:** 1 State Key Laboratory of Oncology in South China, Collaborative Innovation Center for Cancer Medicine, Sun Yat-sen University Cancer Center, Guangzhou, China; 2 MOE Key Laboratory of Gene Function and Regulation, School of Life Sciences, Sun Yat-sen University, Guangzhou, China; 3 First Affiliated Hospital, Sun Yat-sen University, Guangzhou, China; 4 Department of Medical Oncology, Dana-Farber Cancer Institute, Boston, MA USA

**Keywords:** Immunotherapy, Immunotherapy

## Abstract

Overcoming local immunosuppression is critical for immunotherapy to produce robust anti-tumor responses. Myeloid-derived suppressor cells (MDSCs) are key regulators of immunosuppressive networks and promote tumor progression. However, it remains unclear whether and how tumor-infiltrating MDSCs are shaped in response to anti-PD-1 treatment and what their impact on therapeutic efficacy is in colorectal cancer (CRC). In this study, the levels of infiltrating MDSCs were significantly higher in the non-responding organoids and were selectively reduced in the responding group, with MDSCs showing increased apoptosis and attenuated functional activity after anti-PD-1 treatment. A negative correlation between T-cell activation and MDSC function was also observed in fresh human CRC tissues. Mechanistic studies revealed that autocrine IFN-α/β upregulated TRAIL expression on activated T cells to elicit MDSC apoptosis via the TRAIL–DR5 interaction and acted synergistically with TNF-α to inhibit MDSC function of suppressing the T-cell response through the JNK-NMDAR-ARG-1 pathway. Moreover, blockade of IFN-α/β and TNF-α abolished the therapeutic efficacy of anti-PD-1 treatment by preserving the frequency and suppressive activity of infiltrating MDSCs in a CRC mouse model. This result suggested that reprogramming MDSCs by IFN-α/β and TNF-α from activated T cells was necessary for successful anti-PD-1 treatment and might serve as a novel strategy to improve the response and efficacy of anticancer therapy.

## Introduction

Myeloid-derived suppressor cells (MDSCs) are a heterogeneous population of immature myeloid cells that universally regulate the immune response under many pathological conditions.^1–3^ These cells often accumulate in tumors, playing a crucial role in the immunosuppressive tumor microenvironment (TME). MDSCs inhibit the anti-tumor reactivity of T cells and natural killer cells. Furthermore, they promote tumor cell survival, angiogenesis, and metastasis, and affect virtually all cancer therapies, thus emerging as a potential therapeutic target.^4–8^ Although much is known about the roles and underlying mechanisms of MDSC-facilitated tumor progression, significantly less is known about the environmental factors that regulate MDSC generation and maintenance in human tumor tissues.

Immune checkpoint blockade (ICB) treatments have produced promising clinical effects across various tumor types.^9^ ICB aims to reverse the exhausted phenotype of tumor-infiltrating lymphocytes (TILs) by blocking the PD-1/PD-L1 pathway, which is induced to restrict immune activation.^10,11^ Clinical studies have confirmed that ICB treatments elicit potent cytotoxic T-cell-mediated anti-tumor immune responses.^12,13^ In addition to the direct effect on TILs, recent studies reported that anti-PD-1 treatment could also drive tumor-infiltrating dendritic cells to produce interleukin (IL)-12 and CXCL9, which in turn further strengthened T-cell activity and improved cancer immunotherapy.^14,15^ These observations suggested that successful ICB is not solely dependent on the anti-tumor activities of T cells but also requires their interaction with other immune cells. Considering the importance of the myeloid response in tumor pathophysiology, it would be crucial to characterize the influences of T-cell reactions on myeloid remodeling in the TME after anti-PD-1 treatment and their impact on therapeutic efficacy.

Colorectal cancer (CRC) progression is often associated with chronic inflammation^16,17^ and the infiltration of cytotoxic and memory T cells in CRC tissues could be a useful marker to predict good prognosis.^18–20^ CRC with mutations in the mismatch repair pathway, which are associated with increased TIL infiltration, could be more responsive to ICB, but the general benefits remain limited.^21–24^ Recently, we observed that several human tumor tissues, including CRC, are enriched with MDSCs. The selective targeting of tumor-infiltrating MDSCs could improve the efficacy of anti-PD-L1 treatment in a mouse model.^4^ However, it remains unclear whether and how the remodeled TME after ICB could regulate the accumulation and function of MDSCs in CRC tissues.

In the present study, we showed that the response to anti-PD-1 treatment and induction of T-cell activation are associated with the apoptosis and function of infiltrating MDSCs in patient-derived tumor organoids. Mechanistic studies using in vitro models and clinical sample analysis demonstrated that autocrine type I interferons (IFNs) upregulate TNF-related apoptosis-inducing ligand (TRAIL) expression on activated T cells to elicit MDSC apoptosis via the TRAIL–DR5 interaction and act synergistically with tumor necris factor-α (TNF-α) to inhibit the suppressive function of MDSCs through the JNK-NMDAR-ARG-1 pathway. The blockade of type I IFNs and TNF-α retained the frequency and suppressive activity of tumor-infiltrating MDSCs and impaired the therapeutic efficacy of anti-PD-1 treatment in a CRC mouse model. Therefore, reprogramming immunosuppressive myeloid cells might provide a novel therapeutic strategy to improve the responses to anti-PD-1 therapy in CRC.

## Results

### Induction of T-cell activation by an anti-PD-1 mAb is associated with altered MDSC function in PDOs

**Fig. 1:**
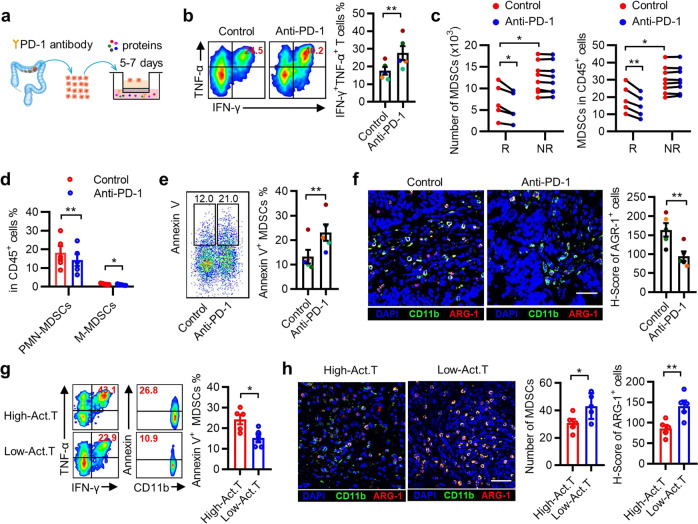
Induction of T-cell activation by anti-PD-1 monoclonal antibody (mAb) is associated with altered MDSC function in patient-derived tumor organoids. **a** Illustration depicting patient-derived tumor organoid (PDO) cultures from colorectal cancer (CRC) tissues, PD-1 antibody (20 μg/mL), and IgG control antibody (20 μg/mL). **b** Representative flow cytometry and statistical analysis of the activation of infiltrated T lymphocytes in organoid CRC tissues after anti-PD-1 treatment. The data shown are from anti-PD-1 therapy-responsive samples (*n* = 5). **c** Quantification of MDSCs in CRC tissues after PD-1 blockade therapy. R indicates that patients responded to anti-PD-1 therapy (*n* = 5) and NR indicates that patients had no response to anti-PD-1 therapy (*n* = 8). **d** Summarized data of the proportion of PMN-MDSCs (CD45^+^Lin^−^HLA-DR^low/^^−^CD33^+^CD11b^+^CD15^+^CD14^−^) and M-MDSCs (CD45^+^Lin^−^HLA-DR^low/^^−^CD33^+^CD11b^+^CD15^−^CD14^+^) in CRC tissues. **e** Annexin V expression on MDSCs was determined using fluorescence-activated cell sorting (FACS) in CRC tissues. **f** Representative immunofluorescence staining and statistical analysis of ARG-1 expression in CD11b^+^ cells in situ in CRC tissues. The data shown in **d**–**f** are from anti-PD-1 therapy-responsive samples (*n* = 5). **g**, **h** Correlation between the number of TNF-α^+^IFN-γ^+^ T cells and the proportion of apoptotic MDSCs and ARG-1 expression in CD11b^+^ cells in fresh CRC tissues (*n* = 10). The scale bar indicates 50 μm. Act. T represents activated T cells. The data are shown as the mean ± SEM. *p < 0.05; **p < 0.01

To evaluate the effect of anti-PD-1 immunotherapy on MDSCs in human CRC tissues, we used an air–liquid interface patient-derived tumor organoid (PDO) model.^25^ The organoids from 13 patients with CRC were treated with a function-blocking PD-1 monoclonal antibody (mAb) or control IgG for 5–7 days (Fig. 1a). A 20% increase in the frequencies of TNF-α^+^IFN-γ^+^ T lymphocytes in the PD-1 mAb group compared with the matched IgG control group was considered a response to anti-PD-1 treatment, which was observed in 5 of the 13 samples (Fig. 1b and Supplementary Fig. S1a). Both the number and frequency of MDSCs in CD45^+^ cells isolated from tumor organoids were significantly higher in the non-responsive group than in the responsive group (Fig. 1c). After treatment with anti-PD-1 mAb, MDSC levels (including both polymorphonuclear (PMN)-MDSCs and mononuclear (M)-MDSCs) were selectively reduced in the tumor organoids from the responsive group but remained unchanged in the non-responsive group (Fig. 1d and Supplementary Fig. S1b). These data indicated that tissue-infiltrated MDSCs are associated with the anti-PD-1 treatment response.

We then assessed the functional status of MDSCs in these tumor organoids. As illustrated in Fig. 1e and Supplementary Fig. S1c, treatment with PD-1 mAb markedly increased MDSC apoptosis and decreased the in situ expression of arginase 1 (ARG-1, a suppressive molecule of MDSCs) in the CD11b^+^ myeloid cells (Fig. 1f and Supplementary Fig. S1d) of anti-PD-1 therapy-responsive patients. Moreover, in freshly resected CRC tissues, high levels of activated T cells were associated with increased MDSC apoptosis and reduced MDSC numbers and ARG-1 expression (Fig. 1g, h). Thus, the status of MDSCs correlated closely with the response to anti-PD-1 therapy in CRC tissues.

### Activated T cells effectively induce MDSC apoptosis and inhibit the suppressive function of MDSCs in vitro

**Fig. 2:**
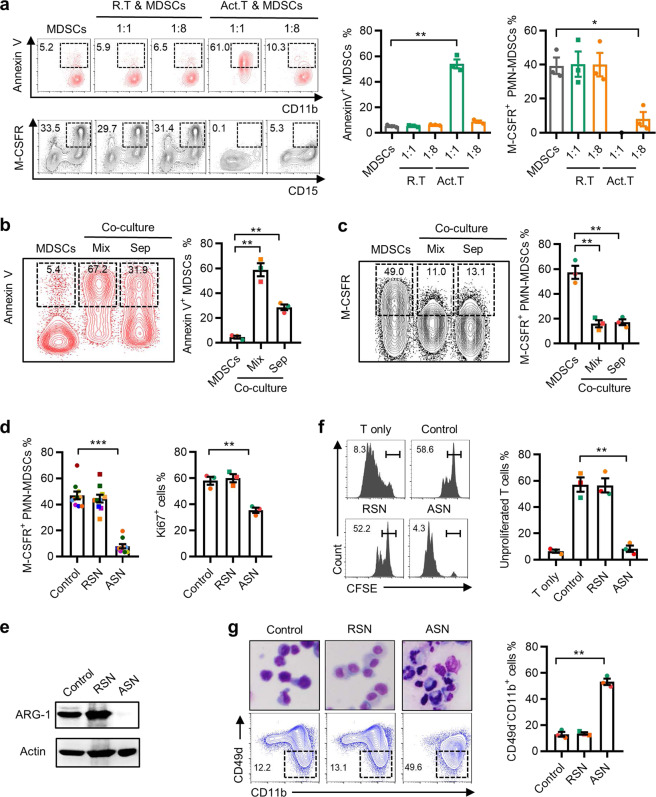
Activated T cells effectively induce apoptosis and inhibit the suppressive function of MDSCs in vitro. **a** CD34^+^ progenitor cells isolated from cord blood were treated with GM-CSF and G-CSF for 3 days to induce MDSCs and then cocultured with resting T cells (R. T) or activated T cells (Act. T) at different ratios. **b**, **c** FACS analysis of MDSCs cultured in medium or with activated T cells directly or in a Transwell system at a ratio of 1 : 1 (for Annexin V detection) or 8 : 1 (for M-CSFR detection) for 3 days. **d**–**g** MDSCs were treated with 10% supernatant from rest T cells (RSN) or activated T cells (ASN) for 3 days. The expression of M-CSFR, Ki-67 (**d**) and ARG-1 (**e**), and the suppression capacity (**f**) and maturity (**g**) of MDSCs was monitored. The data are representative of three independent experiments (**a**–**c**, **d** (right), **e**–**g**) or ten independent experiments (**d**, left). The data are shown as the mean ± SEM. *p < 0.05; **p < 0.01; ***p < 0.001

To elucidate the mechanisms underlying the impaired suppressive function of activated T-cell-educated MDSCs, we used human cord blood-derived haematopoietic progenitors and established a short-term culture system to induce MDSCs in vitro.^4,26^ These induced MDSCs were cocultured with different ratios of T cells that had been activated by CD3 and CD28 antibodies, and recombinant IL-2 for 3 days. Coculture with activated T cells elicited a dose-dependent increase in apoptotic MDSCs (Fig. 2a and Supplementary Fig. S2a). At a ratio of 1 : 1 (activated T cells : MDSCs), more than half of the MDSCs (54.03 ± 6.2%) were positively stained with annexin V. Interestingly, coculture at a lower T-cell ratio (1 : 8), which induced only marginal MDSC apoptosis, still caused a dramatic decrease (from 39.3 ± 8.4% to 8 ± 7.2%) in the expression of macrophage colony-stimulating factor receptor (M-CSFR, a key component in the activation of the MDSC suppressive program) in MDSCs. In addition, apoptotic MDSCs had no inhibitory effect on T-cell proliferation or the generation of TNF-α^+^IFN-γ^+^ T cells (Supplementary Fig. S2b–d).

We then attempted to determine the mechanisms by which activated T cells regulate MDSC activity. Transwell assays showed that the cytotoxic effect of T cells on MDSCs largely relied on cell–cell contact (Fig. 2b). However, the reduced expression of M-CSFR on MDSCs was largely unaffected by Transwell separation, indicating that activated T-cell-secreted soluble compounds might block MDSC activity (Fig. 2c). Thus, we incubated MDSCs with supernatants from activated T cells or resting T cells. Consistently, incubation with activated T-cell supernatant (ASN) resulted in a marked downregulation of the proliferation, immune-suppressive activity, and M-CSFR and ARG-1 expression of MDSCs (Fig. 2d–f). Moreover, ASN also induced PMN-MDSC (CD11b^+^CD49d^−^) maturation (Fig. 2g). Collectively, these data indicated that intercellular contact is required to induce MDSC apoptosis when cultured with a high ratio of activated T cells, while the suppressive function of MDSCs could be hampered by soluble mediators derived from activated T cells, even at a low ratio.

### Activated T cells promote MDSC apoptosis through the TRAIL–TRAILR pathway

**Fig. 3:**
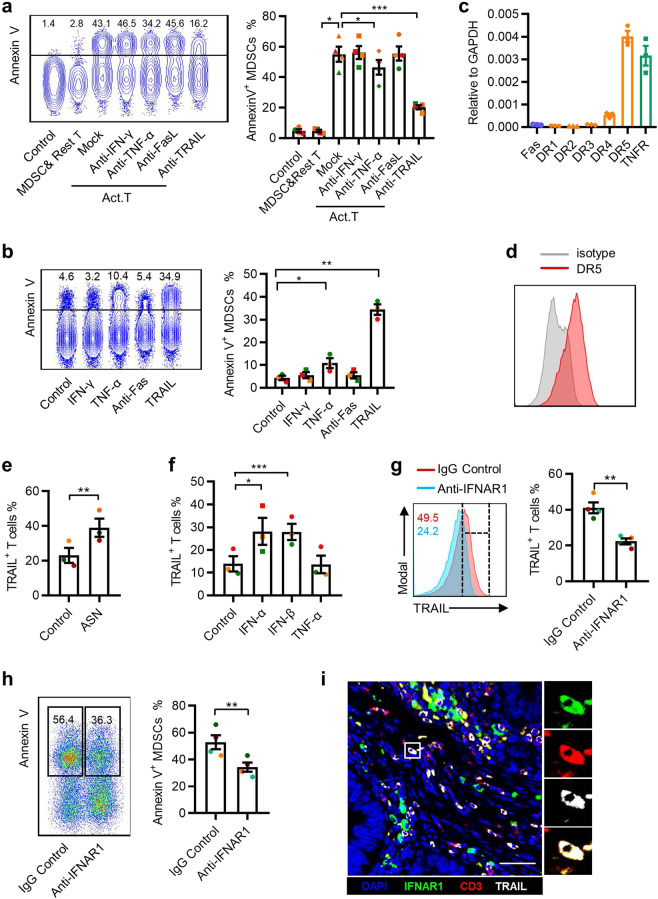
Activated T cells promote MDSC apoptosis through the TRAIL–TRAILR pathway. **a** MDSCs were cocultured with T cells and mAbs against the indicated blocking antibodies and Annexin V expression on MDSCs was determined using FACS. **b** MDSCs were incubated for 48 h with rhTNF-α, rhINF-γ, rhTRAIL, or Fas agonist antibodies, and Annexin V expression on MDSCs was determined using FACS. **c** Apoptosis-related receptors were examined in MDSCs using qPCR. **d** Surface DR5 expression on MDSCs was measured using FACS. The data are representative of three subjects. **e** TRAIL^+^ T cells were determined 48 h following exposure to 15% ASN and CD3/28 antibodies (2 μg/mL). T cells in the control group were only treated with CD3/28 antibodies. **f** Type I interferons (10 ng/mL) and TNF-α (10 ng/mL) were added to the activated T-cell culture system and the expression of TRAIL was determined using FACS. **g**, **h** T cells were incubated for 48 h with CD3/28 antibodies (5 μg/mL) and mAbs against IFNAR1 (2 μg/mL) or the control Ab (IgG1, 10 μg/mL). The expression of TRAIL was analyzed using FACS (**g**); T cells were cocultured with MDSCs for 48 h and Annexin V expression was detected on MDSCs (**h**). **i** The expression of TRAIL (gray), CD3 (red), and IFNAR1 (green) in CRC tissues was determined using confocal microscopy. DAPI staining appears blue. Scale bar = 50 μm. Images in **i** are representative of five subjects. The data were generated from three (**b**–**f**) or four (**a**, **g**, **h**) independent experiments. The data are shown as the mean ± SEM. *p < 0.05; **p < 0.01; ***p < 0.001

T-cell-mediated apoptosis acts mainly through the granzyme/perforin, TNF-α/IFN-γ, and TRAIL/FasL pathways.^27,28^ We used blocking antibodies for these pathways and observed that the neutralization of TRAIL in the coculture system significantly reduced the fraction of Annexin V^+^ MDSCs (from 57.1 ± 9.9% to 20.3 ± 2.9%; Fig. 3a), whereas the blockade of IFN-γ, TNF-α, and FasL had little effect. To further support the role of TRAIL in the induction of MDSC apoptosis, recombinant human (rh) IFN-γ, TNF-α, TRAIL, or Fas agonists were added individually to MDSC culture systems. Only rhTRAIL markedly increased the proportion of Annexin V^+^ MDSCs (Fig. 3b). Furthermore, real-time PCR to determine the expression of TRAIL receptors on MDSCs indicated high expression levels of the mRNA of TRAIL death receptors (*DR4*, *DR5*) (Fig. 3c). High levels of DR5 protein were further confirmed using flow cytometry (Fig. 3d).

Consequently, we measured the levels of TRAIL expression in T cells. Significantly increased TRAIL expression was observed in CD3/CD28-activated T cells and in T cells that had been cultured for 48 h with ASN (Fig. 3e and Supplementary Fig. S3a). Activated T cells produce large amounts of type I IFN and TNF-α (Supplementary Fig. S3b); therefore, we examined their relative contribution to TRAIL expression: IFN-α and IFN-β significantly induced TRAIL expression, while TNF-α barely had an impact (Fig. 3f). Analogously, blocking the type I IFN pathway using anti-IFNAR1 markedly reduced TRAIL expression on activated T cells and impaired their ability to induce MDSC apoptosis (Fig. 3g, h). Multiple immunofluorescence staining showed that TRAIL^+^ T cells expressed IFNAR1 in in situ tumor sections from patients with CRC (Fig. 3i). Overall, these data demonstrated that autocrine type I IFNs could upregulate TRAIL expression on activated T cells, which in turn induced MDSC apoptosis via the TRAIL–DR5 interaction.

### IFN-α/β and TNF-α secreted by activated T cells cooperate to inhibit MDSC function

**Fig. 4:**
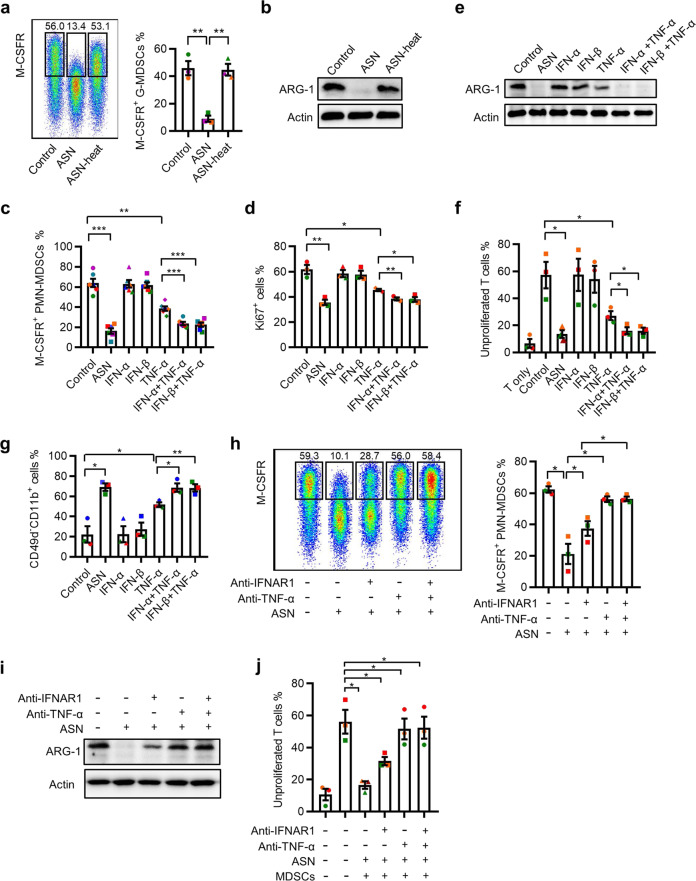
IFN-α/β and TNF-α secreted by activated T cells cooperate to inhibit MDSC function. **a**, **b** The expression of M-CSFR (**a**) and ARG-1 (**b**) of MDSCs in the presence of ASN or denatured ASN, which was removed by heating, was determined using FACS (**a**) or western blotting (**b**). **c**–**g** MDSCs were treated with IFN-α (0.5 ng/mL), IFN-β (0.5 ng/mL), TNF-α (2 ng/mL), or their combination. The expression of M-CSFR (**c**), Ki-67 (**d**), and ARG-1 (**e**) was estimated at day 3; the cells were then cocultured with CFSE-labeled T cells for 6 days. The suppressive activity of MDSCs was monitored using FACS (**f**). The percentage of CD11b^+^CD49d^−^ cells was analyzed using FACS (**g**). **h**–**j** MDSCs were exposed to ASN with control IgG1 (10 μg/mL), TNF-blocking mAbs (2 μg/mL), IFNAR1 mAbs (2 μg/mL), or their combination for 3 days. The levels of M-CSFR and ARG-1 in MDSCs were determined using FACS (**h**) and western blotting (**i**); the suppressive capacity of T cells was measured using FACS (**j**). Immunoblots are representative of 3 subjects (**b**, **e**, **i**). The data were generated from three independent experiments (**a**, **d**, **f**, **g**, **h**, **j**) or six independent experiments (**c**). The data are shown as the mean ± SEM. *p < 0.05; **p < 0.01; ***p < 0.001

Next, we investigated the mechanisms by which soluble substances from activated T cells regulate MDSC function (Fig. 2c). The heat denaturation of proteins from ASN almost completely abolished its capacity to inhibit M-CSFR and ARG-1 expression on MDSCs (Fig. 4a, b). Considering the upregulation of type I IFN and TNF-α in activated T cells (Supplementary Fig. S3b), we wondered whether these two cytokines were sufficient to inhibit MDSC activity. Treatment with TNF-α significantly decreased (64.0%, 50.94–78.80% vs. 38.6%, 31.0–47.4%) M-CSFR expression in MDSCs, whereas IFN-α or IFN-β alone had no effect (Fig. 4c). However, the combination of IFN-α or IFN-β with TNF-α synergistically reduced M-CFR expression (64.0%, 50.94–78.80% vs. 23.6%, 19.8–30.9% vs. 22.5%, 15.1–28.8%; Fig. 4c). The roles of TNF-α and type I IFN in MDSC function were further supported by analyzing their effects on the proliferation, immunosuppressive activity, and maturation of MDSCs (Fig. 4d–g). To further determine the effect of TNF-α and type I IFN on MDSCs, specific neutralizing antibodies were used to antagonize their functions. Consistent with the results using recombinant cytokines, the blockade of IFNAR1 signaling alone partially attenuated the immunosuppressive activity of MDSCs; however, the neutralization of TNF-α significantly antagonized the effects of ASN on MDSCs (Fig. 4h–j). These findings suggested that type I IFN and TNF-α secreted by activated T cells synergistically inhibit MDSC function.

### Activated T cells suppress MDSC function through the JNK-NMDAR-ARG-1 pathway

**Fig. 5:**
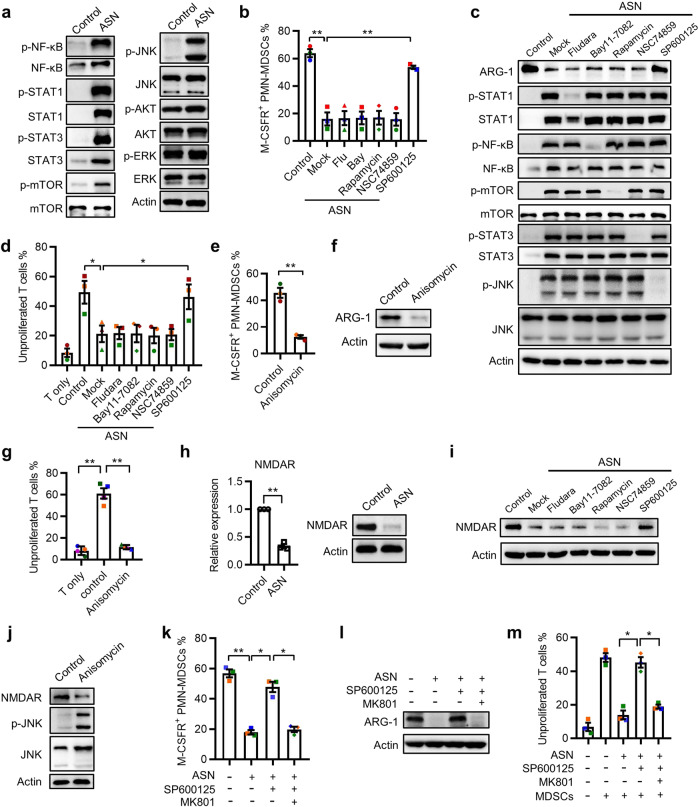
Activated T cells suppress MDSC function through the JNK-NMDAR-ARG-1 pathway. **a** The levels of p-NF-κB, NF-κB, p-STAT1, STAT1, p-STAT3, STAT3, p-mTOR, mTOR, p-JNK, JNK, p-AKT, ATK, p-ERK, and ERK were examined using western blotting in MDSCs stimulated with ASN for 3 days. **b**–**d** MDSCs were treated with ASN and the STAT1 inhibitor Fludara (10 μg/mL), the NF-κB inhibitor Bay 11–7082 (5 μg/mL), the mTOR inhibitor rapamycin (5 μg/mL), the STAT3 inhibitor NSC 74859 (5 μg/mL), or JNK inhibitors (5 μg/mL) for 3 days. The expression of M-CSFR (**b**) and ARG-1 (**c**), and the suppressive capacity (**d**) of MDSCs were assessed. **e**–**g** MDSCs were left untreated or stimulated with the JNK agonist anisomycin (1 μg/mL) and the expression of M-CSFR (**e**) and ARG-1 (**f**) and the suppressive capacity (**g**) of MDSCs were measured. **h** The mRNA and protein levels of NMDAR in MDSCs were determined following exposure to ASN. **i**, **j** NMDAR expression on MDSCs in the presence of ASN combined with the indicated inhibitors (**i**) or the JNK agonist anisomycin (**j**) alone was examined using western blotting. **k**–**m** The phenotype and suppressive activity of MDSCs were determined after stimulation with ASN in the presence or absence of JNK inhibitors or the NMDAR inhibitor MK801 (50 μmol/L). Immunoblots are representative of 3 subjects (**a**, **c**, **f**, **h**, **i**, **j**, **l**). The data were generated from three independent experiments (**b**, **d**, **e**, **h**, **k**, **m**) or four independent experiments (**g**). The data are shown as the mean ± SEM. *p < 0.05; **p < 0.01

To further explore the signaling mechanisms by which ASN modulates MDSC function, we investigated the roles of nuclear factor-κB (NF-κB), signal transducer and activator of transcription 1 (STAT1), STAT3, c-Jun N-terminal kinase (JNK), mammalian target of rapamycin (mTOR), AKT, and extracellular signal-regulated kinase (ERK), all of which are members of the downstream signaling pathways of type I IFNs and TNF-α. ASN induced a significant increase in NF-κB, STAT1, and STAT3 levels and upregulated NF-κB, STAT1, STAT3, JNK, and mTOR phosphorylation in MDSCs; however, it had no effect on AKT and ERK activation (Fig. 5a). To test which of these activated signals is involved in MDSC function, various irreversible inhibitors were used in our culture system. Only JNK inhibition using SP600125 completely abrogated the ASN-mediated downregulation of MDSC function (Fig. 5b–d). Moreover, JNK pathway activation using a specific agonist (anisomycin) abolished the immunosuppressive capacity of MDSCs (Fig. 5e–g). These results suggested that activated T cells regulate MDSC function via JNK signaling.

The functional status of MDSCs is regulated by the TME. Glutamate metabolism is necessary and sufficient to support the generation of functional MDSCs in human tumor tissues.^4^ Therefore, we examined the expression of *N*-methyl-d-aspartate receptor (NMDAR), a key glutamate receptor for MDSC activation, in our culture system. ASN treatment markedly decreased NMDAR expression on MDSCs, which was abolished by JNK pathway blockade (Fig. 5h, i). Consistently, JNK pathway activation using anisomycin decreased NMDAR expression on MDSCs (Fig. 5j), indicating that NMDAR is a downstream factor in JNK signaling. To further support the role of NMDAR in MDSC regulation, MDSCs were exposed to ASN with SP600125 and MK801 (an NMDAR inhibitor). NMDAR signaling blockade counteracted the recovery of MDSCs’ immunosuppressive activity (Fig. 5k–m). In situ examination of NMDAR distribution showed that MDSCs express high levels of NMDAR in human CRC tissue sections (Supplementary Fig. S4a). These results suggested that ASN inhibits MDSC function through JNK-NMDAR-ARG-1 signaling.

### Association of tumor-infiltrating activated T cells with MDSCs in patients with CRC

**Fig. 6:**
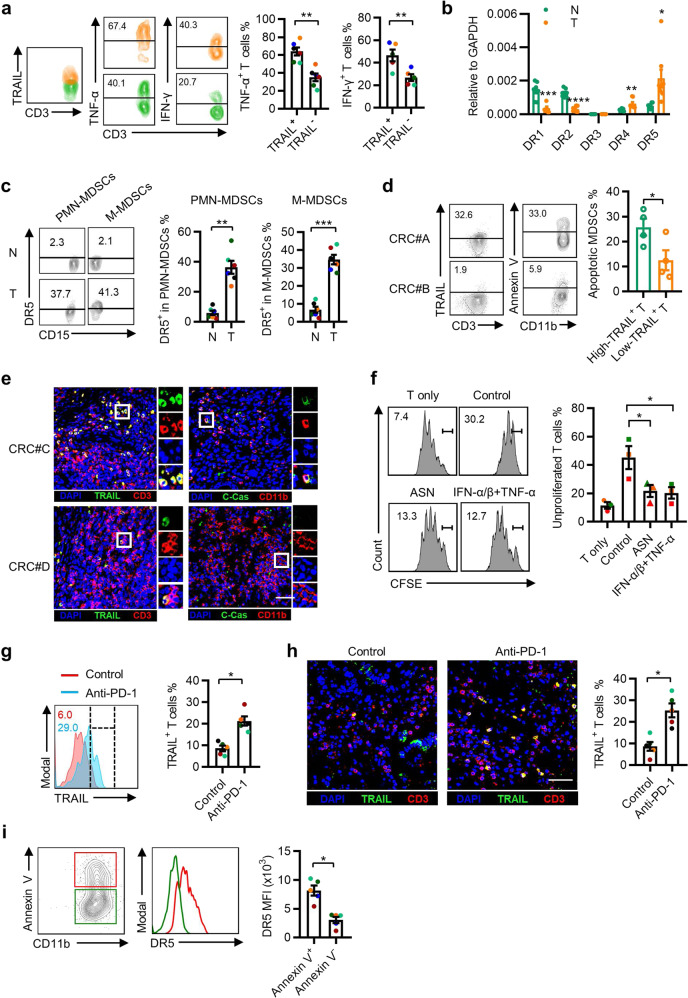
Association of tumor-infiltrating activated T cells with MDSCs in patients with CRC. **a** Representative plot and statistical analysis of IFN-γ and TNF-α levels in TRAIL^+^ or TRAIL^−^ T cells isolated from freshly resected CRC tissues (*n* = 5). **b** The mRNA levels of *DR1*–*DR5* in CD11b^+^ myeloid cells that were purified from non-tumor and tumor regions of CRC tissues were examined by qPCR (*n* = 8). **c** Representative plot and summary of FACS analysis of DR5 expression on PMN-MDSCs and M-MDSCs from paired non-tumor and tumor regions of CRC tissues (*n* = 6). **d**, **e** FACS and immunofluorescence were used to analyze the association of the intra-tumoral apoptotic MDSC frequency with TRAIL^+^ T-cell number. Annexin V^+^ MDSCs and cleaved-Caspase 3^+^ MDSCs both represented apoptotic MDSCs (*n* = 8). **f** CD11b^+^ myeloid cells isolated from CRC tissues were treated ex vivo with ASN, type I interferons and TNF-α, or DMEM for 2 days and then cocultured with CFSE-labeled T cells. FACS was used to examine the proliferation of T cells. (*n* = 3). **g**, **h** Frequencies of TRAIL^+^ T cells in the CRC organoid model after anti-PD-1 therapy were quantified using FACS (**g**) and immunofluorescence (**h**). The data shown are from five CRC samples that showed a response to anti-PD-1 treatment. **i** Representative plot and statistical analysis of DR5 expression in Annexin V^+^ or Annexin V^−^ MDSCs. The data are from five responders to PD-1 blockade therapy. The data are shown as the mean ± SEM. *p < 0.05; **p < 0.01; ***p < 0.001; ****p < 0.0001

We next investigated whether TRAIL, type I IFN, and TNF-α contribute to MDSC status in human CRC tissues. Similar to the in vitro results, infiltrating TRAIL^+^ T cells expressed higher levels of IFN-γ and TNF-α, indicating the upregulation of TRAIL on activated T cells in CRC tissues (Fig. 6a). DR5 expression was significantly upregulated on CD11b^+^ myeloid cells and on MDSCs isolated from fresh tumor tissues compared with their counterparts from paired non-tumoral tissues (Fig. 6b, c). Annexin V^+^ MDSCs expressed higher DR5 levels (Supplementary Fig. S5a). Correlation analysis revealed that TRAIL expression on T cells was positively associated with MDSC death in human CRC tissues (Fig. 6d, e).

To validate the role of type I IFN and TNF-α on tumor-infiltrating MDSCs, CD11b^+^ myeloid cells from fresh CRC tissues were treated with IFN-α/β and TNF-α, and then cocultured with 5 (6)-Carboxyfluorescein diacetate succinimidyl ester (CFSE)-labeled T cells. IFN-α/β and TNF-α markedly impaired the suppressive capacity of MDSCs (Fig. 6f). T cells in tumor tissues exhibited a relatively lower level of TRAIL expression than T cells in non-tumor regions (Supplementary Fig. S5b, c). However, PD-1 mAb treatment in the PDO model significantly upregulated TRAIL expression on infiltrated T cells (Fig. 6g, h and Supplementary Fig. S5d) and Annexin V^+^ MDSCs expressed higher DR5 levels (Fig. 6i and Supplementary Fig. S5e) in CRC patients, in response to PD-1 blockade therapy. These results suggested that activated T cells could promote MDSC death and hamper MDSC function in CRC tissues.

### Blockade of the IFN-α/β and TNF-α pathways reduces the efficacy of anti-PD-1 therapy

**Fig. 7:**
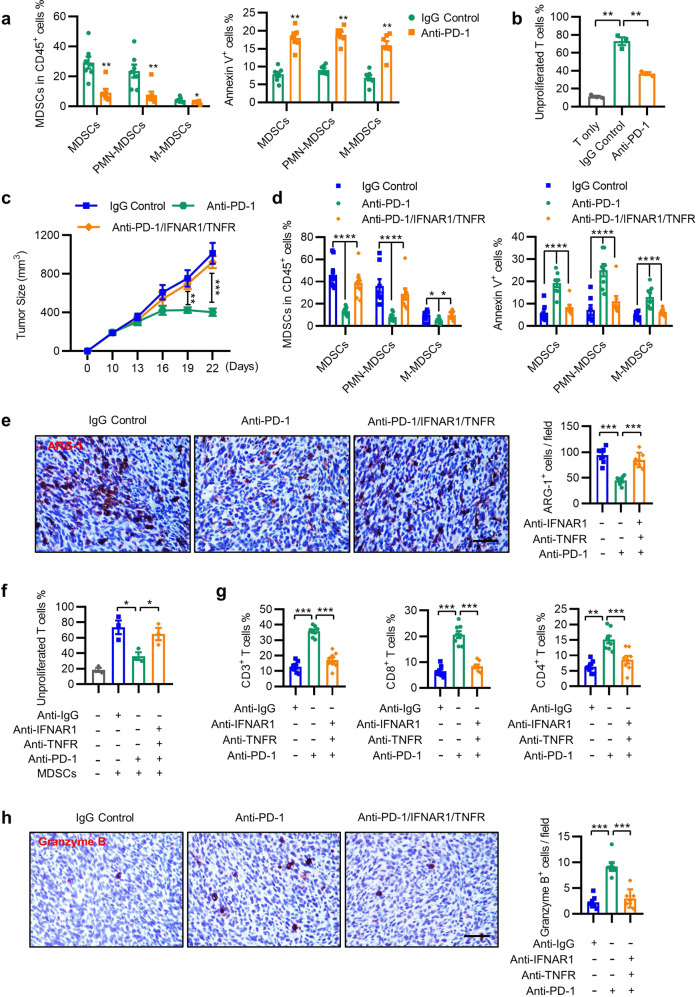
Blockade of the IFN-α/β and TNF-α pathways reduces the efficacy of anti-PD-1 therapy. **a**, **b** CD45^+^ leukocytes from the tumor tissue of MC38 mice were isolated to detect the MDSC number (left) and Annexin V expression (right). The control animals received 100 μg of isotype control antibody, and the experimental group was treated with 100 μg of PD-1 blockade antibody twice a week for 2 weeks (**a**). Tumor-infiltrating myeloid suppressors were further purified using CD11b microbeads and cocultured with CFSE-labeled splenocytes for 3 days in the presence of anti-CD3 (2.5 μg/mL) and anti-CD28 (5 μg/mL) antibodies. The proliferation of splenocytes was determined using FACS (**b**). **c** Mean tumor volumes of subcutaneous MC38 tumors in mice treated with control (100 μg) or PD-1 mAb (100 μg) in combination with anti-IFNAR1 mAb (100 μg) and anti-TNFR mAb (100 μg) twice a week for 2 weeks. **d** Frequencies of tumor-infiltrating MDSCs and Annexin V^+^ MDSCs from the tumor tissues described in **c**. **e**, **f** The expression of ARG-1 (**e**) and the suppressive ability (**f**) of MDSCs in tumor tissues from control and anti-PD-1-treated mice, in combination with anti-IFNAR1 and anti-TNFR therapy, were detected. Scale bar = 50 μm. **g**, **h** The percentages of CD3^+^, CD8^+^, and CD4^+^ T cells determined using FACS (**g**) and the number of granzyme B^+^ cells examined using IHC (**h**) in tumor tissues from the indicated groups are summarized. Scale bar = 50 μm. For the data in **b**, **f**, *n* = 3; for the data in **a**, **e**, **h**, *n* = 7; and for the data in **c**, **d**, **g**, *n* = 8. The data are from two independent experiments. The data are shown as the mean ± SEM. *p < 0.05; **p < 0.01; ***p < 0.001

To confirm the role of reprogramming MDSCs by activated T cells in vivo, we tested the effect of PD-1 mAb on the survival and function of tumor-infiltrating myeloid cells in an ICB-responsive MC38 tumor model. Consistent with the results of the organoid model, anti-PD-1 therapy downregulated the number of MDSCs in CD45^+^ cells, induced MDSC apoptosis, and attenuated their immune-suppressive activity in MC38 tumors (Fig. 7a, b). Immunofluorescence staining revealed that PD-1 mAb treatment increased the number of TRAIL^+^ T cells, coincident with increased apoptosis, and reduced NMDAR expression on the myeloid cells of tumors (Supplementary Fig. S6a).

We then conducted in vivo blockade experiments of both IFN-α/β and TNF-α in the MC38 tumor model. The therapeutic efficacy of PD-1 mAb on tumor growth was almost completely abolished by co-administration of neutralizing Abs against IFNAR1 and TNFR (Fig. 7c and Supplementary Fig. S6b–d). We then examined the numbers and functional statuses of tumor-infiltrating MDSCs and T cells in MC38 tumors. Neutralizing IFNAR1 and TNFR antibodies markedly attenuated the therapeutic effects elicited by PD-1 mAb, including the reduction in the numbers of MDSCs and in ARG-1 expression, the suppressive activity of myeloid suppressors, and the induction of MDSC apoptosis (Fig. 7d–f). Moreover, anti-PD-1 treatment markedly increased the percentage of CD4^+^, CD8^+^, and granzyme B^+^ T cells in tumors, which was markedly inhibited by the blockade of IFNAR1 and TNFR (Fig. 7g, h and Supplementary Fig. S6e). Thus, the production of type I IFNs and TNF-α by activated T cells is necessary to achieve their anti-tumor effects during successful anti-PD-1 treatment by remodeling MDSCs.

## Discussion

Despite the unprecedented durable response rates observed with ICB therapy, efficacy is limited in most patients by a fundamental barrier: the immunosuppressive TME. The results of the present study showed that tumor-infiltrating MDSCs are critical determinants of the CRC tumor response to PD-1 blockade. The induction of T-cell activation by anti-PD-1 was associated with a reduction in the frequency and functional activity of MDSCs in PDOs. Autocrine type I IFNs from activated T cells upregulate T-cell TRAIL expression to induce MDSC apoptosis and act synergistically with TNF-α to suppress MDSC function. Moreover, blockade of IFN-α/β and TNF-α abolished the efficacy of anti-PD-1 treatment by preserving the frequency and suppressive activity of infiltrating MDSCs in a CRC mouse model. These results suggested that reprogramming MDSCs via TNF-α and IFN-α/β might serve as an effective therapeutic strategy to improve the responses to immunotherapy.

Effective anti-tumor responses by ICB are not mediated by directly relieving T-cell anergy alone but rather involve crosstalk between multiple components in the TME. It has been recently shown that the ability of anti-PD-1 to drive sustained tumor control requires a subset of tumor-infiltrating dendritic cells. These dendritic cells produced IL-12 upon sensing the IFN-γ released from T cells, which in turn promoted effector T-cell responses in both mice and patients with cancer.^14^ The present study provides evidence that the activated T-cell reprogramming of MDSCs is essential for the response to anti-PD-1 treatment in CRC tumors. This conclusion is supported by the following observations. First, MDSC levels in the tumor organoids were selectively reduced in the response group, with increased apoptosis and decreased ARG-1 expression in MDSCs after anti-PD-1 treatment. Second, activated T cells effectively induced MDSC apoptosis and inhibited MDSC activity in vitro. Accordingly, high activated T-cell levels were associated with increased MDSC apoptosis, reduced cell numbers, and decreased ARG-1 expression in MDSCs in freshly resected human CRC tissues. Third, in an ICB-responsive mouse model, the anti-tumor effect of PD-1 blockade was associated with reduced numbers and impaired suppressive activity of MDSCs, coincident with increased MDSCs and cytotoxic T-cell apoptosis in MC38 tumors. These effects were almost completely abolished by the blockade of type I IFNs and TNF-α, two key mediators of the apoptosis and suppressive function of MDSCs by activated T cells. Therefore, regulating the crosstalk between MDSCs and T cells might represent a novel therapeutic strategy to improve ICB responses.

MDSCs are key regulators of the immunosuppressive TME and impact virtually all types of cancer therapy. We and others have recently shown that selectively targeting MDSCs was sufficient to improve the therapeutic efficacy of ICB in various tumor-bearing mice.^1,29^ In the present study, autocrine cytokines from activated T cells could effectively induce MDSC apoptosis and suppress MDSC function. Autocrine IFN-α/β upregulated TRAIL expression on T cells and promoted MDSC apoptosis via cell–cell contact mediated by the TRAIL–TRAILR interaction. This hypothesis was supported by a recent study showing the critical role of the TRAIL–TRAILR pathway in promoting MDSC apoptosis by ER stress.^30^ Several other factors, including Fas/FasL, TNF-α, and IFN-γ, are also implicated in T-cell-mediated cytotoxic effects.^31,32^ However, neither Fas/FasL nor IFN-γ were involved in inducing MDSC apoptosis in the current study, and TNF-α demonstrated only a marginal effect. Moreover, apoptotic Treg cells sustain and amplify their suppressor activity to abolish the therapeutic efficacy of ICB.^33^ Apoptotic MDSCs completely lost their suppressive capacity.

Interestingly, coculture with a low ratio of activated T cells was sufficient to attenuate the proliferation and immunosuppressive capacity of MDSCs and promote their maturation. This process was primarily mediated by TNF-α, acting synergistically with IFN-α/β released from activated T cells. Previous studies have shown that TNF-α and IFN-α/β have dual effects on MDSC infiltration and tumor progression in different model systems.^32,34–36^ Our data suggested that type I IFNs and TNF-α cooperatively suppress MDSC function in CRC. Although the precise signaling mechanisms remain unclear, we provided evidence that these two cytokines attenuate MDSC function via JNK activation. The generation of functional MDSCs is controlled by glutamine metabolism in human cancers via the glutamate-NMDA receptor axis.^4^ In the present study, activated T cells downregulated NMDAR expression, which was regulated by JNK activation. The blockade of NMDAR counteracted the recovery of the immunosuppressive activity of MDSCs by the JNK inhibitor. These data revealed a novel regulatory mechanism by which activated T-cell supernatants hampered MDSC function through the JNK-NMDAR-ARG-1 axis.

Several features of genomic instability, such as microsatellite instability (MSI), chromosomal instability and CpG island methylator phenotype, have emerged as major predictive markers for the efficacy of ICB.^37,38^ CRC patients with MSI-high tumors tend to have a good response to anti-PD-1 therapy, with enriched CD8^+^ T cells and activated dendritic cells as well as a higher expression of neoantigens in MSI-high tumors than in MSI-low tumors.^37,39,40^ These findings indicate that immune cell infiltration in tumors is a critical determinant for the clinical response to ICB. We also analyzed the correlation between the MSI phenotype and the response to anti-PD-1 treatment in our PDO models. The results showed that treatment response was found in 83.3% (5/6) of the MSI-high tumors, and no response was observed in MSI-low tumors (Supplementary Table S2). These results, together with our finding that higher densities of infiltrating MDSCs in the non-responsive group, suggested that MDSCs might serve as an important factor in the tumor resistance to anti-PD-1 treatment in MSI-low patients. In addition, the responders had earlier TNM stages and smaller tumor sizes than the non-responders (Supplementary Table S2).

Overcoming the impact of tolerized conditions is critical for robust immunotherapy-induced anti-tumor responses.^41–44^ Given the pre-existing immunosuppressive TME, boosting the activation of cytotoxic immune responses remains a challenge. We found that the non-responsive PDOs contained significantly higher infiltrating MDSC levels, suggesting that MDSCs might form a barrier against the initiation of anti-tumor responses by anti-PD-1 therapy. This hypothesis was supported by our observation that abrogating the generation of functional MDSCs improved the therapeutic efficacy of anti-PD-L1 in an ICB-resistant mouse tumor model.^4^ Other interventions, including type I IFNs and TNF-α, might also help provide favorable conditions to form a positive feedback loop for T-cell activation in ICB therapy.

In summary, we described a previously unrecognized feature of activated T cells in reprogramming MDSCs and indicate its important role in amplifying the responses to ICB therapy. MDSCs are increasingly being recognized as therapeutic targets.^45–47^ Therefore, determining the mechanisms that selectively modulate the accumulation of functional MDSCs could provide a novel strategy to increase the response and efficacy of anticancer therapy by overcoming the pre-existing tolerized TME and inducing a positive feedback loop for T-cell activation in ICB therapy.

## Materials and methods

### Human subjects

CRC samples were obtained from patients who underwent resection at Sun Yat-sen University Cancer Center. All patients were pathologically confirmed without previous anticancer therapy and individuals with concurrent autoimmune disease, HIV, or syphilis were excluded. Paired fresh tumor and non-tumor tissues were used to isolate tissue-infiltrating leukocytes and for immunofluorescence staining and ex vivo culture. Peripheral blood and cord blood samples were obtained from healthy blood donors attending the Guangzhou Blood Center or the First Affiliated Hospital of Sun Yat-sen University, all of whom were negative for antibodies against hepatitis C virus, hepatitis B virus, HIV, and syphilis. All samples were anonymously coded in accordance with local ethical guidelines, as stipulated by the Declaration of Helsinki, with written informed consent obtained from all participants and a protocol approved by the Review Board of the participating institutions.

### Human PDO culture

Primary tumor tissues were cultured as described previously.^25^ One milliliter of reconstituted collagen solution (Trevigen, catalog number 3410-010-01) was added to the Transwell insert (0.4 μm) and solidified for 30 min in a 37 °C incubator under sterile conditions, serving as a bottom layer gel without tissue. The tumor tissues were washed twice in ADMEM/F12 (Invitrogen, catalog number A1370801) containing 1× Normocin (InvivoGen, catalog number ant-nr-2) and 10 × penicillin/streptomycin (Invitrogen, catalog number 15140122) and then minced into 125–500 mm^3^ fragments, resuspended in 1 mL of type I collagen gel, and layered on top of pre-solidified collagen gel (1 mL) to form the double dish air–liquid culture system. The Transwell insert containing tumor tissue and collagen was placed into an outer 60 mm cell culture dish containing 1.0 mL of ADMEM/F12 supplemented with 50% Wnt3a, RSPO1, Noggin-conditioned media with HEPES (1 mM, Sigma, catalog number H4034), glutamine (1×, Gibco, catalog number A2916801), gastrin (10 nM, Sigma, catalog number 05-23-2301), *N*-acetylcysteine (1 mM, Sigma, catalog number A0737), epidermal growth factor (50 ng/mL, R&D Systems, catalog number 236-EG), IL-2 (6000 IU/mL, R&D Systems, catalog number 1081-IL), granulocyte colony-stimulating factor (G-CSF) (50 ng/mL, R&D Systems, catalog number 214-CS-025), and granulocyte macrophage colony-stimulating factor (GM-CSF) (50 ng/mL, R&D Systems, catalog number 215-GM-010). PD-1 antibody (20 μg/mL, MSD, catalog number 1374853-91-4) or IgG control antibody (20 μg/mL, R&D Systems, catalog number AB-108-C) was added to the culture system. Samples were collected after 5 days.

### Generation of MDSCs

Leukocyte cells were isolated from healthy cord blood using Ficoll density centrifugation. CD34^+^ progenitor cells were purified using magnetic beads (Miltenyi Biotec, catalog number 130-046-702) and expanded as previously described.^26^ In brief, CD34^+^ cells were plated at a density of 4 × 10^5^ cells/mL in six-well plates (Corning) with 2 mL/well HSC expansion media (StemSpan SFEM, Stem Cell Technologies, catalog number 09650) supplemented with 100 ng/mL SCF (R&D Systems, catalog number 255-SC-050), 100 ng/mL FLT-3L (R&D Systems, catalog number 308-FK-025), 100 ng/mL TPO (R&D Systems, catalog number 288-TP-025), and 20 ng/mL IL-3 (R&D Systems, catalog number 203-IL-010). The cells were cultured at 37 °C in 5% CO_2_ for 7 days. The media was changed with fresh media on day 3 and day 5, and the cell density was kept at 4 × 10^5^ cells/mL. To obtain MDSCs, the expanded CD34^+^ cells were plated at a density of 2.5 × 10^5^ cells/well in complete Dulbecco’s modified Eagle’s medium (Life Technologies, catalog number C11995500BT) with 40 ng/mL GM-CSF and 40 ng/mL G-CSF, and cultured in a 5% CO_2_-humidified atmosphere for 3–4 days for use in subsequent experiments.

### Coculture of MDSCs with Pan-T cells

Bulk T cells (including naive T cells and memory T cells) were purified from the peripheral blood of healthy donors using the Pan-T Cell Isolation Kit (Miltenyi Biotechnology, catalog number 130-096-535). These bulk T cells and splenocytes were labeled with 2 μM CFSE (Invitrogen, catalog number 65-0850-84) for 15 min, according to the manufacturer’s instructions, and cultured in RPMI 1640 supplemented with 10% fetal bovine serum, 20 U/mL recombinant IL-2 (Invitrogen, catalog number BMS334), 1 μg/mL coated anti-CD3 (human: eBioscience, catalog number 16-0037-85; mouse: BioLegend, catalog number 100314), and 5 μg/mL soluble anti-CD28 (human: eBioscience, catalog number 16-0289-85; mouse: BioLegend, catalog number 102112). MDSCs derived from progenitor cells or freshly isolated myeloid cells were incubated with pan-T cells or splenocytes for 6 days, and were collected and analyzed by flow cytometry.

### Immunohistochemistry and immunofluorescence

Immunohistochemistry and immunofluorescence were performed as previously described.^4,48^ Paraffin-embedded formalin-fixed samples were cut into 4 µm sections, which were then processed for immunohistochemistry or immunofluorescence. The sections were dewaxed in xylene and rehydrated through a decreasing ethanol series. Subsequently, the sections were placed in 0.3% H_2_O_2_ for 10 min at room temperature to quench endogenous peroxidase. Epitope retrieval was performed by boiling the sections in 10 mM citrate buffer for 10 min. The tissues were stained with anti-granzyme B antibody (dilution 1 : 1000, Abcam, catalog number ab4059) and anti-ARG-1 antibody (dilution 1:200, Cell Signaling Technology, catalog number 93668) overnight at 4 °C. The sections were then stained with the corresponding secondary antibodies, visualized with diaminobenzidine in the Envision System (Dako, catalog number K5007), counterstained with Mayer’s haematoxylin, and mounted with non-aqueous mounting medium.

For immunofluorescence analysis, primary antibodies against CD11b (dilution 1 : 500, Abcam, catalog number ab216445), ARG-1 (dilution 1 : 200, Cell Signaling Technology, catalog number 93668), NMDAR (dilution 1 : 200, Abcam, catalog number ab93610), TRAIL (dilution 1 : 200, Abcam, catalog number ab9959) or cleaved caspase (dilution 1 : 200, Cell Signaling Technology, catalog number 9661 S) were used. Primary antibodies were detected with Alexa Fluor 488 TSA Kits (Invitrogen, catalog number B40953) and Alexa Fluor 555 (Invitrogen, catalog number B40955).

The images were viewed by optical microscopy (OLYMPUS) or scanning confocal microscopy (ZEISS, LSM780) and analyzed with ZEN 2.6 software. All stained slides were evaluated in a blinded fashion by 2 pathologists. H-score = 3× percentage of strongly stained nuclei + 2× percentage of moderately stained nuclei + percentage of weakly stained nuclei, giving a range of 0–300.

### Flow cytometry

Cells were stained with fluorochrome-conjugated Abs according to the manufacturer’s instructions. For surface staining, the cells were prepared and suspended in phosphate buffer saline (PBS) solution or 1× Annexin V (BD Biosciences, catalog number 556547) binding buffer supplemented with 1% heat-inactivated fetal calf serum. To detect Ki-67 in nuclei, the cells were fixed and permeabilized using a reagent from eBioscience (catalog number 00-5523-00). In some experiments, the T cells from tissues or ex vivo culture were stimulated with Leukocyte Activation Cocktail (BD, catalog number 550583) at 37 °C for 5 h. Thereafter, the cells were stained with surface markers, fixed and permeabilized with IntraPrep reagent (Beckman Coulter, catalog number A07803), and then stained with intracellular markers. Data were acquired with a Cytoflex flow cytometer (Beckman Coulter) and analyzed with FlowJo software. The fluorochrome-conjugated Abs used are summarized in Supplementary Table S1.

### Immunoblotting

Immunoblotting was performed as previously described.^49^ Protein was extracted using a protein extraction kit (Thermo Fisher, catalog number 89900) according to the manufacturer’s instructions. SDS-polyacrylamide gel electrophoresis (8% or 10%) was used to separate equal amounts of cellular proteins. The membranes were visualized by using a commercial ECL kit (Millipore, catalog number P90720). The antibodies used in immunoblotting are summarized in Supplementary Table S1.

### Quantitative real-time PCR

Quantitative PCR (qPCR) was performed as previously described.^50^ Total RNA was extracted using TRIzol reagent (Life Technologies, catalog number 15596-018). Equal amounts of total RNA from each sample were subjected to oligo (dT)-primed cDNA synthesis using 5× All-In-One RT MasterMix (Abm, catalog number G492). Reactions were run according to a standard protocol using SYBR Green Realtime MasterMix (TOYOBO, catalog number QPS-201) on a Roche Light Cycler 480 System (Roche Diagnostics). All results are presented in arbitrary units relative to human ACTIN mRNA expression. The specific primers used for qPCR are summarized in Supplementary Table S1.

### Animals and cell lines

Female C57/c mice (6–8 weeks of age) were purchased from Guangdong Medical Laboratory Animal Center. All mice were maintained under specific pathogen-free conditions in the animal facilities of Sun Yat-sen University Cancer Center and all animal experiments were performed according to state guidelines and approved by the Institutional Animal Care and Use Committee of Sun Yat-sen University. MC38 cells were a gift from Rui-Hua Xu (Sun Yat-sen University Cancer Center).

### Tumor models and treatments

MC38 tumor cells (1 × 10^6^) were injected subcutaneously into the flanks of the mice, and tumor growth was monitored for up to 25 days. Tumor dimensions were measured along three orthogonal axes once tumors were palpable with callipers and calculated as tumor volume = abc/2. For the therapeutic anti-PD-1 treatment, 100 μg of anti-PD-1 (Bio X cell, catalog number BE0273) or the corresponding IgG2b isotype control (Bio X cell, catalog number BE0090) in 100 µl of PBS was administered intraperitoneally to the mice four times at 3-day intervals 10 days after tumor cell transplantation. For anti-IFNAR1 and anti-TNFR treatment, the mice received 100 μg of anti-IFNAR1 (Bio X cell, catalog number BE0241) and 100 μg of anti-TNFR (Bio X cell, catalog number BE0274) or vehicle intraperitoneally every 3 days for a total of four times.

### Statistical analysis

All experiments were performed using at least three different samples. The statistical significance of intergroup differences was analyzed by using a two-tailed Student’s *t*-test and all values are expressed as the mean ± SEM. All data were analyzed using IBM SPSS (version 21.0; IBM Corp., Armonk, NY, USA) statistical software. All data were analyzed by using two-tailed tests unless otherwise specified, and *P* < 0.05 was considered statistically significant.

## Supplementary information

Supplementary Information

Supplementary Figure S1

Supplementary Figure S2

Supplementary Figure S3

Supplementary Figure S4

Supplementary Figure S5

Supplementary Figure S6

## Data Availability

All data supporting the findings of this study are available from the corresponding author on reasonable request.

## References

[1] Veglia F, Perego M, Gabrilovich D (2018). Myeloid-derived suppressor cells coming of age. Nat. Immunol..

[2] Kumar V, Patel S, Tcyganov E, Gabrilovich DI (2016). The nature of myeloid-derived duppressor cells in the tumor microenvironment. Trends Immunol..

[3] Bronte V (2016). Recommendations for myeloid-derived suppressor cell nomenclature and characterization standards. Nat. Commun..

[4] Wu WC (2019). Immunosuppressive immature myeloid cell generation is controlled by glutamine metabolism in human cancer. Cancer Immunol. Res..

[5] Wu C (2018). Spleen mediates a distinct hematopoietic progenitor response supporting tumor-promoting myelopoiesis. J. Clin. Investig..

[6] Haas L, Obenauf AC (2019). Allies or enemies-the multifaceted role of myeloid cells in the tumor microenvironment. Front. Immunol..

[7] Jahchan NS (2019). Tuning the tumor myeloid microenvironment to fight cancer. Front. Immunol..

[8] Engblom C, Pfirschke C, Pittet MJ (2016). The role of myeloid cells in cancer therapies. Nat. Rev. Cancer.

[9] Pardoll DM (2012). The blockade of immune checkpoints in cancer immunotherapy. Nat. Rev. Cancer.

[10] Topalian SL, Drake CG, Pardoll DM (2015). Immune checkpoint blockade: a common denominator approach to cancer therapy. Cancer Cell..

[11] Andrews LP, Yano H, Vignali DAA (2019). Inhibitory receptors and ligands beyond PD-1, PD-L1 and CTLA-4: breakthroughs or backups. Nat. Immunol..

[12] Liu D (2019). Integrative molecular and clinical modeling of clinical outcomes to PD1 blockade in patients with metastatic melanoma. Nat. Med..

[13] Tang H (2016). Facilitating T cell infiltration in tumor microenvironment overcomes resistance to PD-L1 blockade. Cancer Cell..

[14] Garris CS (2018). Successful anti-PD-1 cancer immunotherapy requires T cell-dendritic cell crosstalk involving the cytokines IFN-gamma and IL-12. Immunity.

[15] Chow MT (2019). Intratumoral activity of the CXCR3 chemokine system is required for the efficacy of anti-PD-1 therapy. Immunity.

[16] Lasry A, Zinger A, Ben-Neriah Y (2016). Inflammatory networks underlying colorectal cancer. Nat. Immunol..

[17] Terzic J, Grivennikov S, Karin E, Karin M (2010). Inflammation and colon cancer. Gastroenterology.

[18] Nosho K (2010). Tumour-infiltrating T-cell subsets, molecular changes in colorectal cancer, and prognosis: cohort study and literature review. J. Pathol..

[19] Fakih M (2019). Immune overdrive signature in colorectal tumor subset predicts poor clinical outcome. J. Clin. Investig..

[20] Gajewski TF, Schreiber H, Fu YX (2013). Innate and adaptive immune cells in the tumor microenvironment. Nat. Immunol..

[21] Topalian SL (2012). Safety, activity, and immune correlates of anti–PD-1 antibody in cancer. N. Engl. J. Med..

[22] Brahmer JR (2010). Phase I study of single-agent anti–programmed death-1 (MDX-1106) in refractory solid tumors: safety, clinical activity, pharmacodynamics, and immunologic correlates. J. Clin. Oncol..

[23] Yaghoubi N (2019). PD-1/ PD-L1 blockade as a novel treatment for colorectal cancer. Biomed. Pharmacother..

[24] Xie, Y.-H., Chen, Y.-X. & Fang, J.-Y. Comprehensive review of targeted therapy for colorectal cancer. *Sig. Transduct. Target. Ther*. **5**, 22 (2020). 10.1038/s41392-020-0116-z PMC7082344 32296018

[25] Neal JT (2018). Organoid modeling of the tumor immune microenvironment. Cell.

[26] Wu WC (2014). Circulating hematopoietic stem and progenitor cells are myeloid-biased in cancer patients. Proc. Natl Acad. Sci. USA.

[27] Martinez-Lostao L, Anel A, Pardo J (2015). How do cytotoxic lymphocytes kill cancer cells?. Clin. Cancer Res..

[28] Duan S, Thomas PG (2016). Balancing immune protection and immune pathology by CD8(+) T-cell responses to influenza infection. Front. Immunol..

[29] Fleming V (2018). Targeting myeloid-derived suppressor cells to bypass tumor-induced immunosuppression. Front. Immunol..

[30] Condamine T (2014). ER stress regulates myeloid-derived suppressor cell fate through TRAIL-R-mediated apoptosis. J. Clin. Investig..

[31] Sinha P (2011). Myeloid-derived suppressor cells express the death receptor Fas and apoptose in response to T cell-expressed FasL. Blood.

[32] Deng L (2014). Irradiation and anti-PD-L1 treatment synergistically promote antitumor immunity in mice. J. Clin. Investig..

[33] Maj T (2017). Oxidative stress controls regulatory T cell apoptosis and suppressor activity and PD-L1-blockade resistance in tumor. Nat. Immunol..

[34] Stone ML (2017). Epigenetic therapy activates type I interferon signaling in murine ovarian cancer to reduce immunosuppression and tumor burden. Proc. Natl Acad. Sci. USA.

[35] Taleb K (2017). Chronic type I IFN is sufficient to promote immunosuppression through accumulation of myeloid-derived suppressor cells. J. Immunol..

[36] Hu X (2014). Transmembrane TNF-alpha promotes suppressive activities of myeloid-derived suppressor cells via TNFR2. J. Immunol..

[37] Picard E (2020). Relationships between immune landscapes, genetic subtypes and responses to immunotherapy in colorectal cancer. Front. Immunol..

[38] Mandal R (2019). Genetic diversity of tumors with mismatch repair deficiency influences anti-PD-1 immunotherapy response. Science.

[39] Dudley JC (2016). Microsatellite instability as a biomarker for PD-1 blockade. Clin. Cancer Res..

[40] Overman MJ (2017). Nivolumab in patients with metastatic DNA mismatch repair-deficient or microsatellite instability-high colorectal cancer (CheckMate 142): an open-label, multicentre, phage 2 study. Lancet Oncol..

[41] Zou W (2005). Immunosuppressive networks in the tumour environment and their therapeutic relevance. Nat. Rev. Cancer.

[42] Binnewies M (2018). Understanding the tumor immune microenvironment (TIME) for effective therapy. Nat. Med..

[43] Flores CT (2018). Lin-CCR2^+^ hematopoietic stem and progenitor cells overcome resistance to PD-1 blockade. Nat. Commun..

[44] Wang, X. et al. PD-L1 is a direct target of cancer-FOXP3 in pancreatic ductal adenocarcinoma (PDAC), and combined immunotherapy with antibodies against PD-L1 and CCL5 is effective in the treatment of PDAC. *Sig. Transduct. Target. Ther*. **5**, 38 (2020). 10.1038/s41392-020-0144-8 PMC7162990 32300119

[45] Mao Y (2016). Targeting suppressive myeloid cells potentiates checkpoint inhibitors to control spontaneous neuroblastoma. Clin. Cancer Res..

[46] Messmer MN, Netherby CS, Banik D, Abrams SI (2015). Tumor-induced myeloid dysfunction and its implications for cancer immunotherapy. Cancer Immunol. Immunother..

[47] Lecot P (2019). Neutrophil heterogeneity in cancer: from biology to therapies. Front. Immunol..

[48] Xu J (2017). Vascular CXCR4 expression promotes vessel sprouting and sensitivity to sorafenib treatment in hepatocellular carcinoma. Clin. Cancer Res..

[49] Li XF (2015). Increased autophagy sustains the survival and pro-tumourigenic effects of neutrophils in human hepatocellular carcinoma. J. Hepatol..

[50] Kuang DM (2014). B7-H1-expressing antigen-presenting cells mediate polarization of protumorigenic Th22 subsets. J. Clin. Investig..

