# Supplementary material for: Reprogramming immunosuppressive myeloid cells by activated T cells promotes the response to anti-PD-1 therapy in colorectal cancer
Source: Signal Transduct Target Ther. 2021 Jan 8;6:4. doi: 10.1038/s41392-020-00377-3 (PMC7791142; doi:10.1038/s41392-020-00377-3)
Supplement: Supplementary file 1 — Supplementary Information [file 41392_2020_377_MOESM1_ESM.docx]

**Supplementary Materials for**

**Reprogramming immunosuppressive myeloid cells by activated T cells promotes the response to anti-PD-1 therapy in colorectal cancer**

**Running title**: Activated T cells reprogram MDSCs in ICB therapy

Jing Chen^a^, Hong-Wei Sun^a^, Yan-Yan Yang^a,b^, Hai-Tian Chen^c^, Xing-Juan Yu^a^, Wen-Chao Wu^a,d^, Yi-Tuo Xu^a^, Li-Lian Jin ^b^, Xiao-Jun Wu^a^, Jing Xu^a*^ and Limin Zheng^a,b*^

^a^State Key Laboratory of Oncology in South China, Collaborative Innovation Center for Cancer Medicine, Sun Yat-sen University Cancer Center, Guangzhou, P. R. China; ^b^MOE Key Laboratory of Gene Function and Regulation, School of Life Sciences, Sun Yat-sen University, Guangzhou, P. R. China. ^c^First Affiliated Hospital, Sun Yat-sen University, Guangzhou, P. R. China. ^d^Department of Medical Oncology, Dana-Farber Cancer Institute, Boston, United States.

Jing Chen and Hong-Wei Sun contributed equally to this article.

Correspondence to: xujing@sysucc.org.cn and [zhenglm@mail.sysu.edu.cn](mailto:zhenglm@mail.sysu.edu.cn)

**This PDF file includes**

Figures. S1 to S6

Tables S1 to S2

Figures. S1 to S6

Figure. S1.


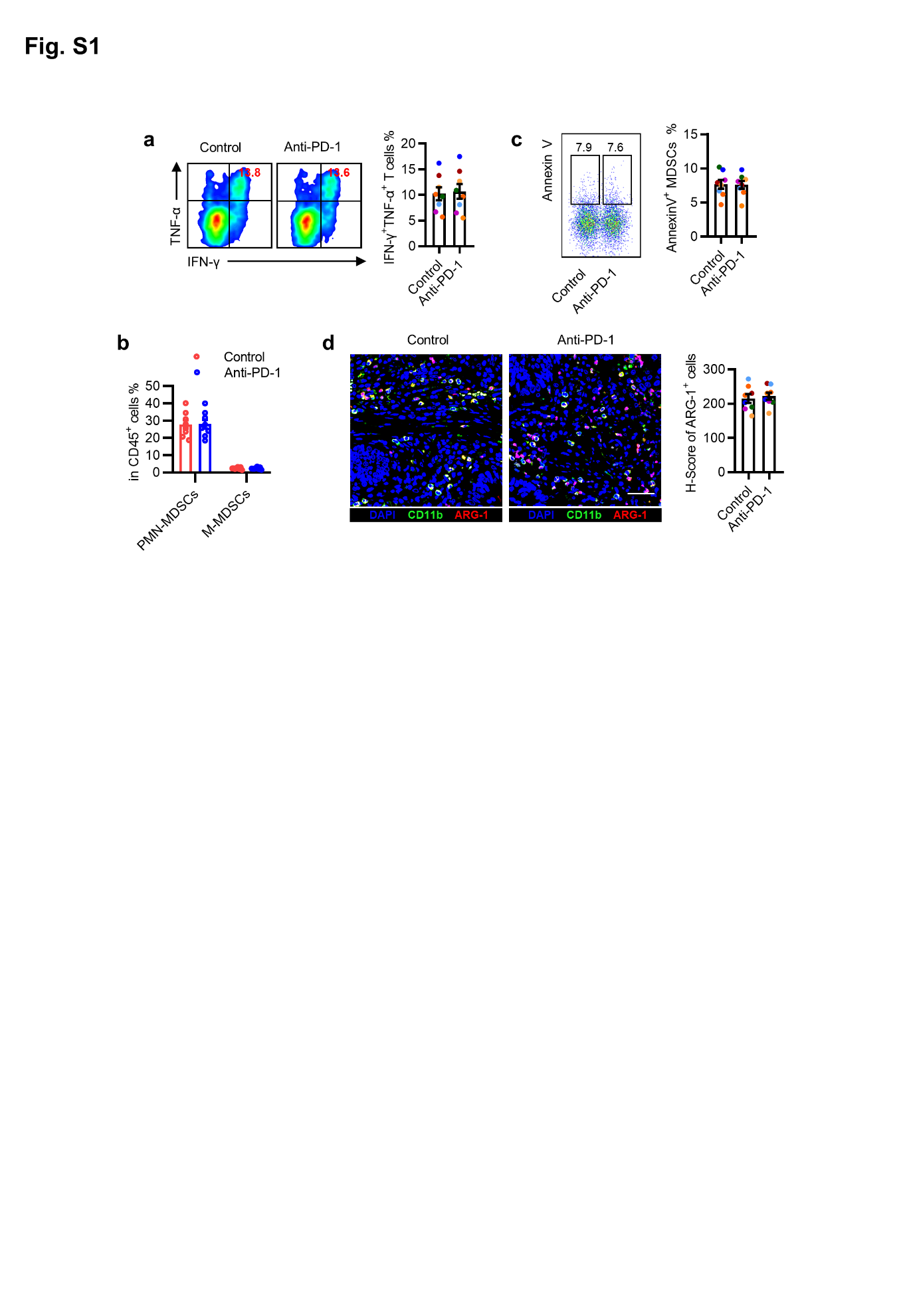


**Fig. S1. The effect of anti-PD-1 treatment on non-responsive CRC organoids.**

The data shown were from 8 CRC samples that had no response to anti-PD-1 therapy. **(a)** Representative flow cytometry and statistical analysis of the activation of infiltrated T lymphocytes in organoid CRC tissues after anti-PD-1 treatment. **(b)** Quantification of MDSCs, PMN-MDSCs and M-MDSCs in CRC tissues after PD-1 blockade. **(c)** Annexin V expression on MDSCs was monitored. **(d)** Representative immunofluorescence staining and statistical analysis of ARG-1 expression on CD11b^+^ cells in situ in CRC tissues after PD-1 blockade in PDO culture. The data are from 8 independent experiments. The data are shown as the mean ± SEM.

Figure. S2.


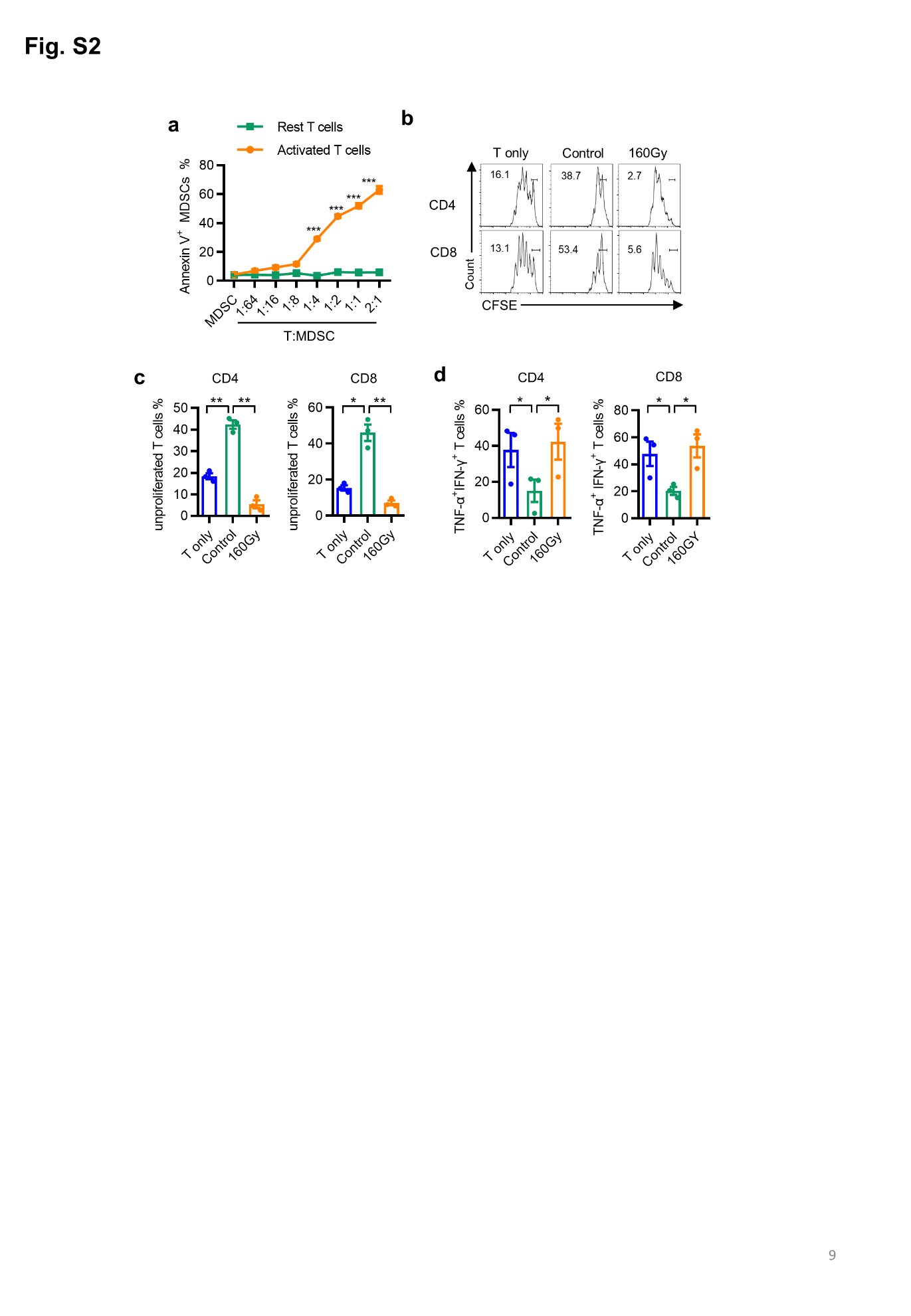


**Figure. S2. Activated T cells promote MDSC apoptosis and further lead to MDSC dysfunction.**

**(a)** MDSCs cocultured with a concentration gradient of resting T cells or activated T cells. MDSC apoptosis was monitored using FACS. **(b-d)** Apoptotic MDSCs were induced by irradiation. CFSE-labeled T cells were cocultured with control or apoptotic MDSCs or medium alone for 3 days. FACS was used to analyze the effect of immunosuppressive activity on T cell proliferation and the cytokine secretion of apoptotic MDSCs. The data are from 3 independent experiments. The data are shown as the mean ± SEM. *p < 0.05; **p < 0.01; ***p < 0.001.

Figure. S3.


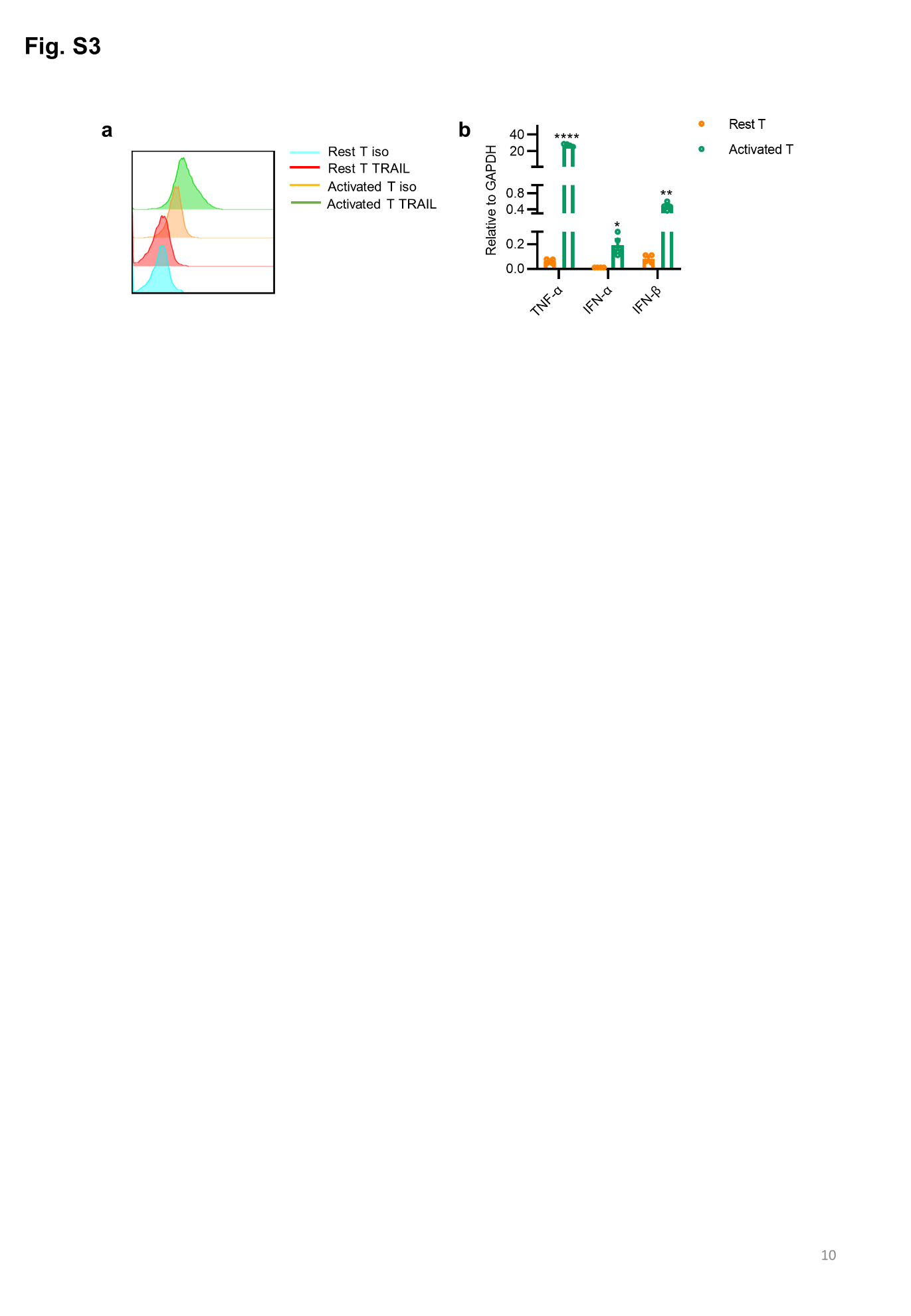


**Fig. S3. The phenotype of activated T cells.**

**(a)** Representative plot of TRAIL expression in resting or activated T cells. The data shown are representative of 3 subjects. **(b)** qPCR was used to detect the expression of type I interferons and TNF-α in resting or activated T cells. The data are from 4 independent experiments. The data are shown as the mean ± SEM. **p* < 0.05; ***p* < 0.01; *****p* < 0.0001.

Figure. S4.


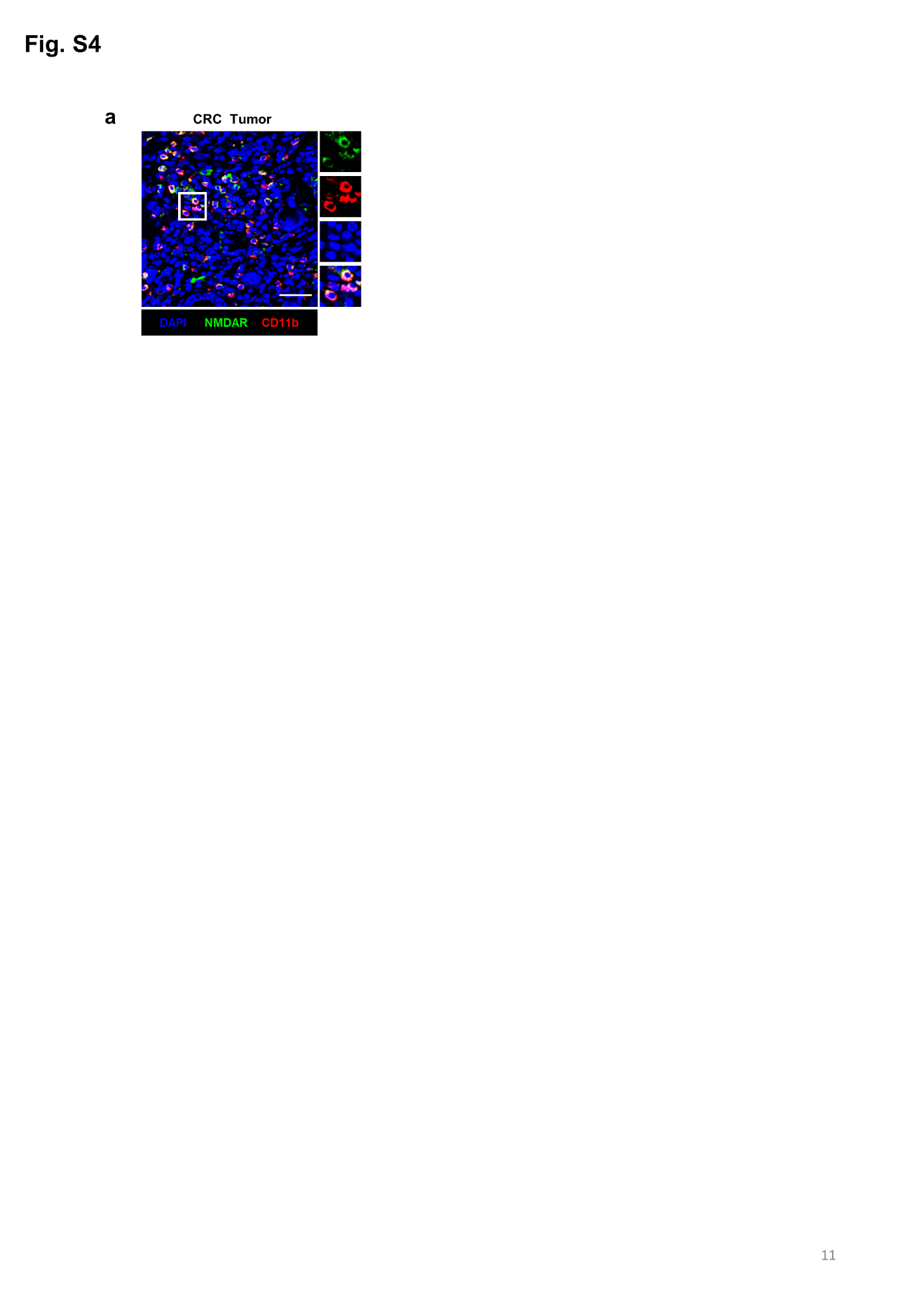


**Fig. S4. MDSCs highly express NMDAR in colorectal cancer.**

**(a)** The expression of NMDAR in myeloid suppressive cells of CRC tissues was analyzed using confocal microscopy. Scale bar = 50 μm. The data shown are representative of 5 subjects.

Figure. S5.


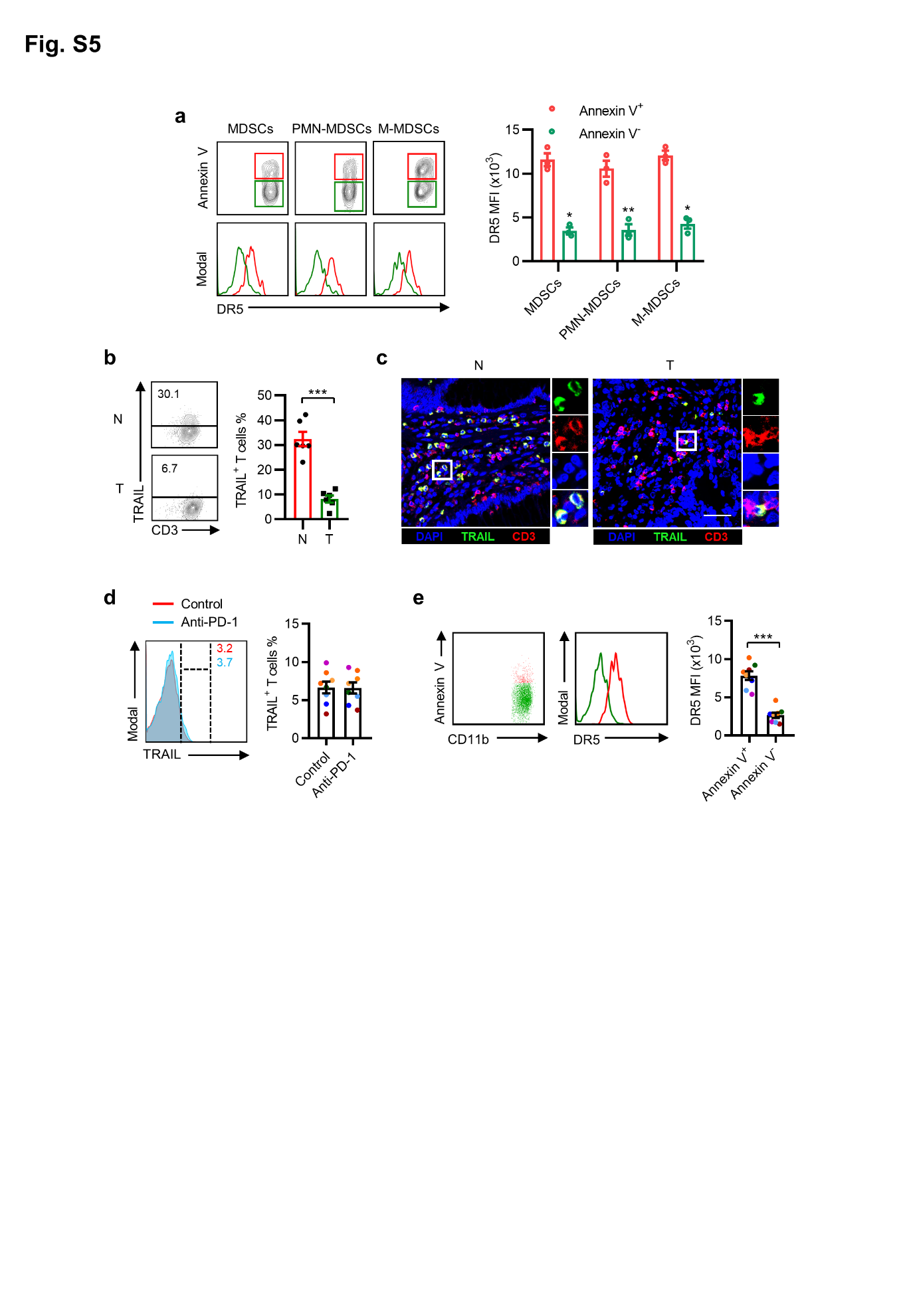


**Fig. S5. The TRAIL-TRAILR pathway exists in colorectal cancer.**

**(a)** Representative plots and statistical analysis of DR5 expression in Annexin V^+^ or Annexin V^-^ MDSCs from fresh CRC tissues (n = 3). **(b)** Representative FACS plot and summary of TRAIL^+^CD3^+^ T cells from CRC tissues (n = 6). **(c)** Multiple immunofluorescence staining of CD3 (red), TRAIL (green), and DAPI (blue) in CRC tissues was analyzed using confocal microscopy. One of six representative micrographs is shown. **(d, e)** Frequencies of TRAIL^+^ T cells in the CRC organoid model were examined by FACS **(d)**; DR5 expression in Annexin V^+^ or Annexin V^-^ MDSCs **(e)**. The data shown are from 8 non-responders to PD-1 blockade therapy **(d，e)**. The data are shown as the mean ± SEM. ****p* < 0.001.

Figure. S6.


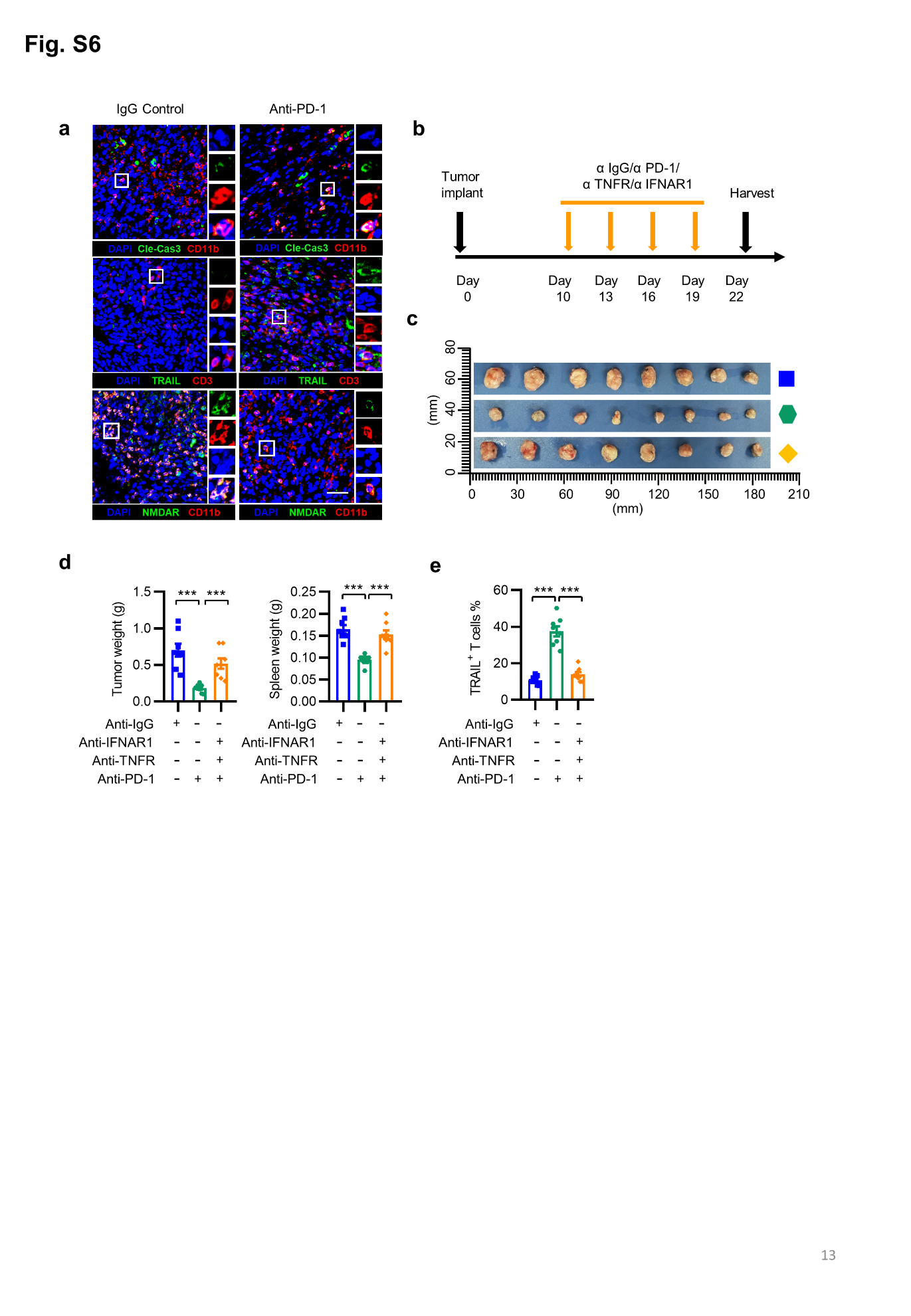


**Figure. S6. Blockade of IFN-α/β and TNF-α impairs anti-PD-1 therapy-mediated tumor regression.**

**(a)** Expression of cleaved-caspase3 (green) in CD11b^+^ cells (red), TRAIL (green) on CD3^+^ cells (red), and NMDAR (green) on CD11b^+^ cells (red) in tissues from the organoid model. One of seven representative micrographs is shown**. (b)** Design of experiments exploring the effects of IFN-α/β and TNF-α on tumor progression. MC38 cells were injected into the flank of wild-type mice, and then PD-1 mAb, IFNAR1 mAb, and TNFR mAb were administered intraperitoneally twice a week for two weeks. **(c, d)** The effect of combination treatments of anti-PD-1, anti-IFNAR1, and anti-TNFR on the tumor volume **(c)**, weight and spleen weight **(d)** (*n* = 8). **(e)** The frequency of TRAIL^+^CD3^+^ T cells isolated from tumor tissues was determined using FACS (*n* = 8). All data shown are from 2 independent experiments. The data are shown as the mean ± SEM. ****p* < 0.001.

Table S1.

**Table S1. Summary of Reagents and Materials**

| **Reagents or Materials** | **Source** | **Cat. No.** |
| --- | --- | --- |
| **Antibodies (Flow Cytometry)** | | |
| CD45-Krome Orange | Beckman Coulter | A96416 |
| CD33-APC | Beckman Coulter | IM2471 |
| CD33-PE | eBioscience | 12-0338 |
| CD3-PC5.5 | eBioscience | 45-0037 |
| CD19-PC5.5 | eBioscience | 45-0199 |
| CD56-PC5.5 | eBioscience | 35-0567 |
| CD11b-PE-Cy7 | Beckman Coulter | A54822 |
| HLA-DR-PE-CF594 | BD Biosciences | 562304 |
| CD14-AF700 | BD Biosciences | 557923 |
| CD15-EF450 | eBioscience | 48-0159 |
| CD15-FITC | eBioscience | 11-0159 |
| CD15-APC | BioLegend | 301907 |
| M-CSFR-APC | R&D Systems | FAB329A-100 |
| DR5-PE | BioLegend | 307405 |
| TRAIL-PE | BioLegend | 308205 |
| Annexin V-FITC | BD Biosciences | 556547 |
| Annexin V-APC | BD Biosciences | 550474 |
| Ly6G-PE-CF594 | BD Biosciences | 562700 |
| CD11b-PE-Cy7 | BD Biosciences | 561098 |
| Ly6C-AF647 | BioLegend | 128009 |
| CD8a-AF700 | BD Biosciences | 557959 |
| CD3-BV421 | BD Biosciences | 562877 |
| CD4-BV421 | BioLegend | 100437 |
| CD45-BV605 | BioLegend | 103140 |
| **Antibodies (Immunoblotting)** | | |
| NMDAR | Abcam | ab9361 |
| Arginase 1 | Cell Signaling Technology | 79404 |
| β-Actin | Cell Signaling Technology | 4970 |
| p-STAT3 | Cell Signaling Technology | 19145 |
| t-STAT3 | Cell Signaling Technology | 12640 |
| p-STAT1 | Cell Signaling Technology | 9167S |
| t-STAT1 | Cell Signaling Technology | 9172S |
| p-P65 | Cell Signaling Technology | 3033T |
| t-P65 | Cell Signaling Technology | 8242T |
| p-mTOR | Cell Signaling Technology | 5536T |
| t-mTOR | Cell Signaling Technology | 2983T |
| p-JNK | Cell Signaling Technology | 4668T |
| t-JNK | Cell Signaling Technology | 9252T |
| p-AKT | Cell Signaling Technology | 9271T |
| t-AKT | Cell Signaling Technology | 3686T |
| p-Erk | Cell Signaling Technology | 4370T |
| t-Erk | Cell Signaling Technology | 4695T |
| **Chemicals** | | |
| MK801 | Apexbio | A3100 |
| Fludara | Apexbio | A8317 |
| Bay11-7082 | Apexbio | A4210 |
| Rapamycin | Apexbio | A8167 |
| NSC74859 | Apexbio | A8338 |
| SP600125 | Apexbio | A4604 |
| **Recombinant protein** | | |
| IFN-α2 | Sino Biological | 13833-HNAY |
| IFN-β | Sino Biological | 10704-HNAS |
| TNF-α | R&D Systems | 210-TA-020 |
| IFN-γ | R&D Systems | 285-IF-100 |
| TRAIL | R&D Systems | 375-TL-010 |
| **Primers** | | |
| Human DCR1 forward primer 5'- CCGTTAGGGAACTCTGGGGA -3' | Thermo Fisher | customized |
| Human DCR1 reverse primer 5'- GGCAGTGGTGGCAGAGTAAG -3' | Thermo Fisher | customized |
| Human DCR2 forward primer 5'- GGATGCTTGCCTCTCCCTATC -3' | Thermo Fisher | customized |
| Human DCR2 reverse primer 5'- CTCGTGAAGGACATGAACGC -3' | Thermo Fisher | customized |
| Human DR3 forward primer 5'- TCACCCTTCTACTGCCAACC -3' | Thermo Fisher | customized |
| Human DR3 reverse primer 5'- CATCCCACGACAGCTAGGAAT -3' | Thermo Fisher | customized |
| Human DR4 forward primer 5'- GTCTGTTGTTGCATCGGCTC -3' | Thermo Fisher | customized |
| Human DR4 reverse primer 5'- AGAGACGAAAGTGGACAGCG -3' | Thermo Fisher | customized |
| Human DR4 forward primer 5'- GTCTGTTGTTGCATCGGCTC -3' | Thermo Fisher | customized |
| Human DR5 reverse primer 5'- CAGTGTGTCAGTGCGAAGAAG -3' | Thermo Fisher | customized |
| Human Fas forward primer 5'- AACACTGTGACCCTTGCACC -3' | Thermo Fisher | customized |
| Human Fas reverse primer 5'- AGAAGACAAAGCCACCCCAAG -3' | Thermo Fisher | customized |
| Human TNFR forward primer 5'- CCAAATGGGGGAGTGAGAGG -3' | Thermo Fisher | customized |
| Human TNFR reverse primer 5'- AGGTGAGGGACCAGTCCAAT -3' | Thermo Fisher | customized |
| Human TNF-α forward primer 5'- TCAGAGGGCCTGTACCTCAT -3' | Thermo Fisher | customized |
| Human TNFα reverse primer 5'- GGAGGTTGACCTTGGTCTGG -3' | Thermo Fisher | customized |
| Human IFN-α1 forward primer 5'- CTTGTGCCTGGGAGGTTGTC -3' | Thermo Fisher | customized |
| Human IFN-α1 reverse primer 5'- AGCAGGGGTGAGAGTCTTTG -3' | Thermo Fisher | customized |
| Human IFN-α2 forward primer 5'- CTTGTGCCTGGGAGGTTGTC -3' | Thermo Fisher | customized |
| Human IFN-α2 reverse primer 5'- AGGTGAGCTGGCATACGAATC -3' | Thermo Fisher | customized |
| Human IFN-β reverse primer 5'- GCGACACTGTTCGTGTTGTC -3' | Thermo Fisher | customized |
| Human IFN-β forward primer 5'- GGCAGTATTCAAGCCTCCCA -3' | Thermo Fisher | customized |
| Human ACTB forward primer 5'- TCACCAACTGGGACGACATG -3' | Thermo Fisher | customized |
| Human ACTB reverse primer 5'- TGATCTGGGTCATCTTCTCGC- 3' | Thermo Fisher | customized |

Table S2.

| **Table S2. Clinicopathological parameters of anti-PD-1 treatment responders and non-responders** | | | | |
| --- | --- | --- | --- | --- |
| Characteristics | No. of patients | Anti-PD-1 treatment | | *p* value |
|  |  | Response | Non-response |  |
| Sex |  |  |  |  |
| Female | 5 | 2 | 3 | 0.685 |
| Male | 8 | 3 | 5 |  |
| Age (years) |  |  |  |  |
| ≤ 60 | 6 | 2 | 4 | 0.587 |
| > 60 | 7 | 3 | 4 |  |
| Tumor differentiation |  |  |  |  |
| Well to moderate | 7 | 3 | 4 | 0.587 |
| Poor | 6 | 2 | 4 |  |
| Tumor size (cm) |  |  |  |  |
| ≤ 5.0 | 5 | 4 | 1 | **0.032** |
| > 5.0 | 8 | 1 | 7 |  |
| TNM stage |  |  |  |  |
| I-III | 5 | 4 | 1 | **0.032** |
| IV | 8 | 1 | 7 |  |
| Primary tumor location |  |  |  |  |
| Right side of the colon | 7 | 3 | 4 | 0.587 |
| Left side of the colon | 6 | 2 | 4 |  |
| Microsatellite instability |  |  |  |  |
| High | 6 | 5 | 1 | **0.005** |
| Low | 7 | 0 | 7 |  |
| Preoperative CEA (ng/ml) |  |  |  |  |
| ≤ 5.0 | 6 | 2 | 4 | 0.587 |
| > 5.0 | 7 | 3 | 4 |  |
| Preoperative CA199 (U/ml) |  |  |  |  |
| ≤ 35.0 | 8 | 3 | 5 | 0.685 |
| > 35.0 | 5 | 2 | 3 |  |
| NOTE: *P* values were analyzed by χ2 test or Fisher’s exact test, as appropriate. Italic values indicate significant *p* values (*p* < 0.05). | | | | |
| Abbreviations: TNM stage: tumor-node-metastasis classification, CEA: Carcinoembryonic antigen, CA199: Carbohydrate antigen 199. | | | | |
