# Supplementary figures and images for: Reprogramming immunosuppressive myeloid cells by activated T cells promotes the response to anti-PD-1 therapy in colorectal cancer
Source: Signal Transduct Target Ther. 2021 Jan 8;6:4. doi: 10.1038/s41392-020-00377-3 (PMC7791142; doi:10.1038/s41392-020-00377-3)

Fig. S1

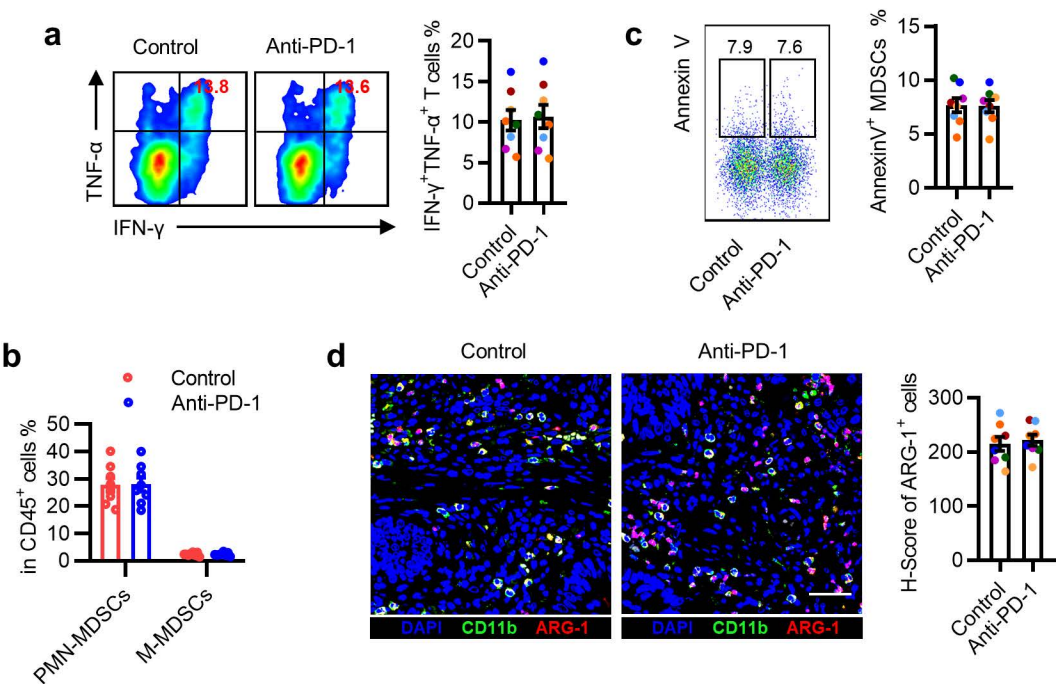

Supplement: Supplementary file 2 — Supplementary Figure S1 [file 41392_2020_377_MOESM2_ESM.pdf]

Fig. S2

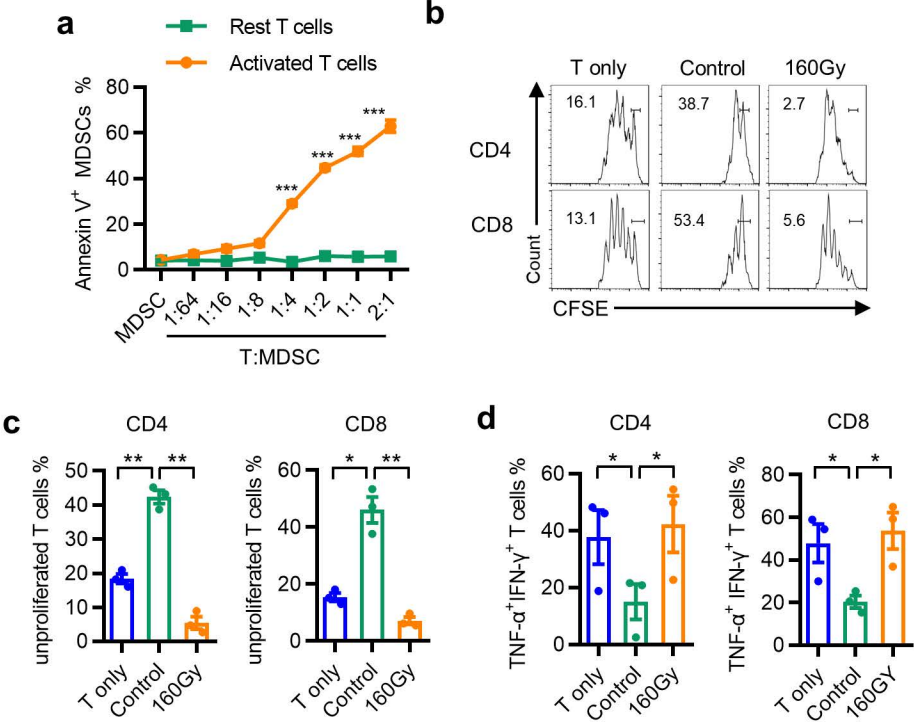

Supplement: Supplementary file 3 — Supplementary Figure S2 [file 41392_2020_377_MOESM3_ESM.pdf]

Fig. S3

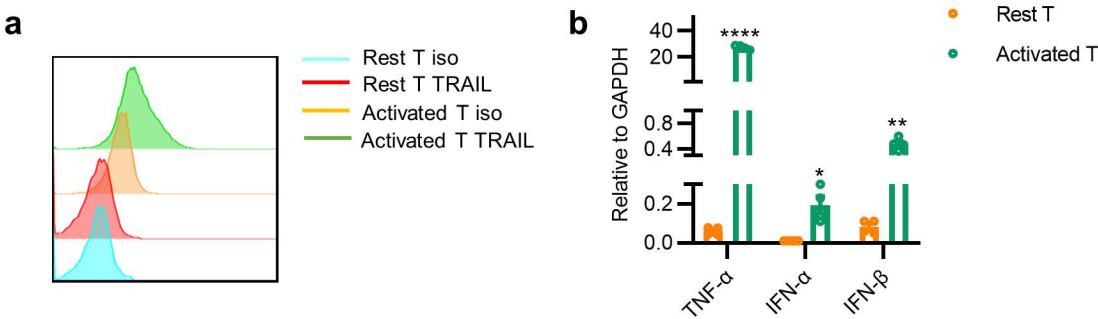

Supplement: Supplementary file 4 — Supplementary Figure S3 [file 41392_2020_377_MOESM4_ESM.pdf]

Fig. S4

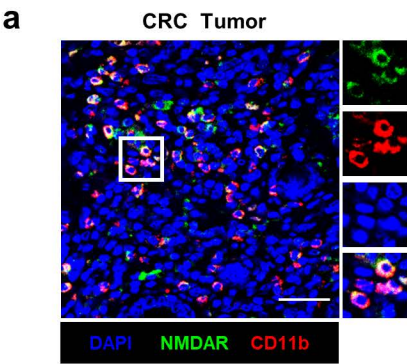

Supplement: Supplementary file 5 — Supplementary Figure S4 [file 41392_2020_377_MOESM5_ESM.pdf]

Fig. S5

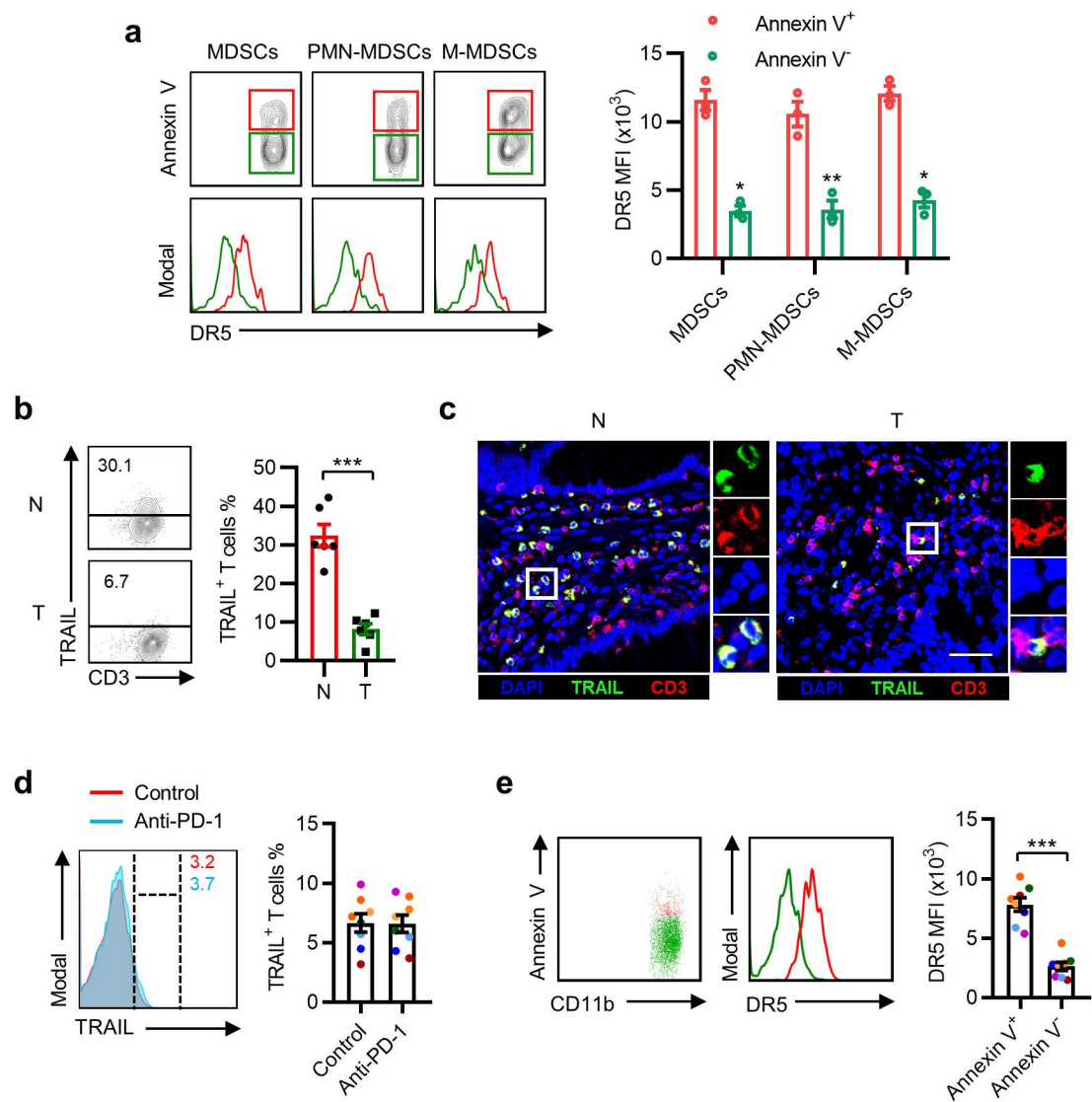

Supplement: Supplementary file 6 — Supplementary Figure S5 [file 41392_2020_377_MOESM6_ESM.pdf]

Fig. S6

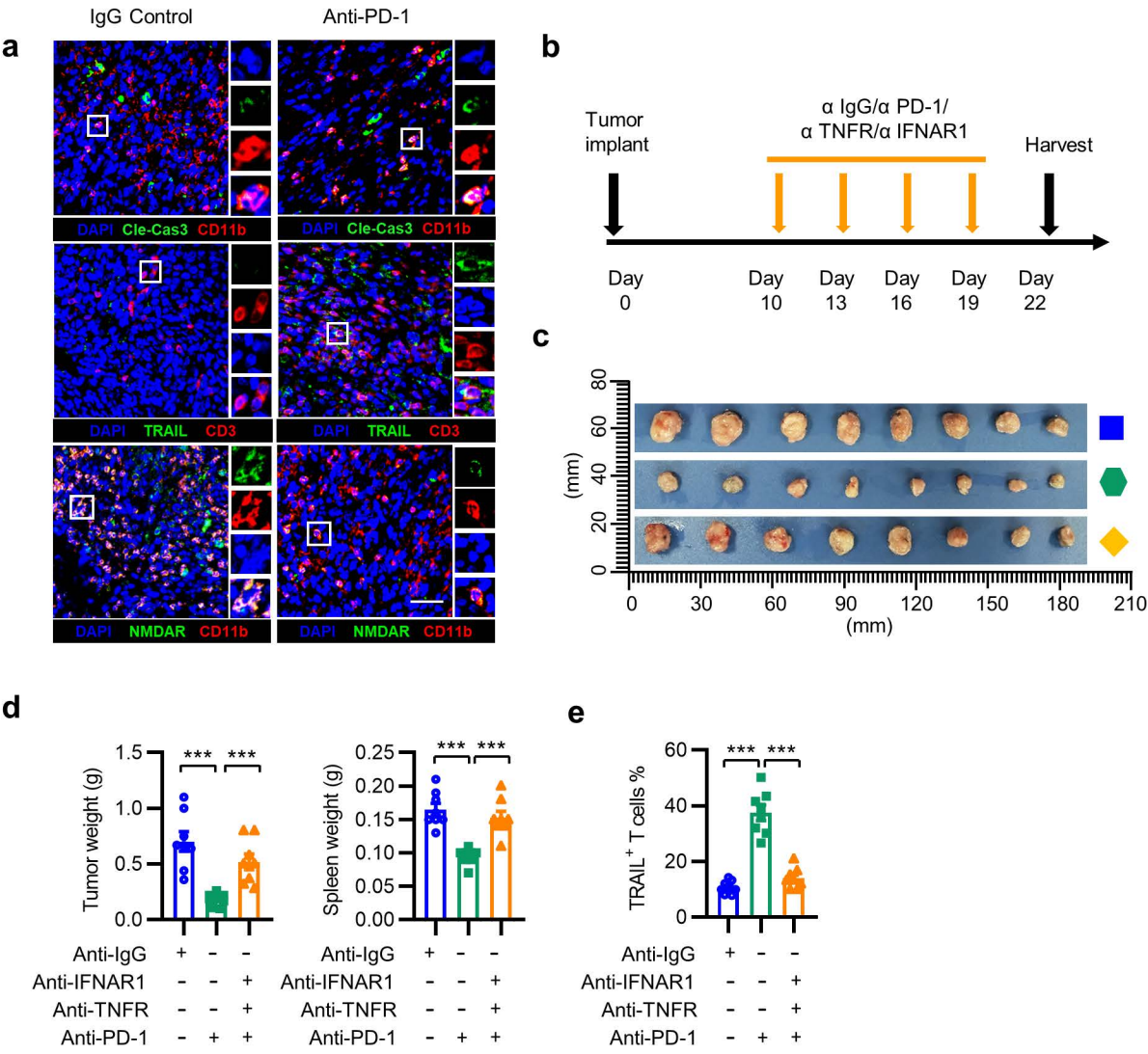

Supplement: Supplementary file 7 — Supplementary Figure S6 [file 41392_2020_377_MOESM7_ESM.pdf]
